# The impact of governance in primary health care delivery: a systems thinking approach with a European panel

**DOI:** 10.1186/s12961-019-0456-8

**Published:** 2019-07-04

**Authors:** Ana Belén Espinosa-González, Brendan C. Delaney, Joachim Marti, Ara Darzi

**Affiliations:** 1 Centre for Health Policy, IGHI, Department of Surgery and Cancer, Imperial College London, Room 1035, 10th Floor Queen Elizabeth Queen Mother Wing, St Mary’s Hospital South Wharf Road, London, W2 1NY United Kingdom; 2 Department of Surgery and Cancer, Imperial College London, London, United Kingdom; 3 Centre for Primary Care and Public Health (Unisanté), University of Lausanne, Lausanne, Switzerland

**Keywords:** Delphi study, framework, governance, decentralisation, financing, regulation, primary health care

## Abstract

**Background:**

Enhancing primary health care (PHC) is considered a policy priority for health systems strengthening due to PHC’s ability to provide accessible and continuous care and manage multimorbidity. Research in PHC often focuses on the effects of specific interventions (e.g. physicians’ contracts) in health care outcomes. This informs narrowly designed policies that disregard the interactions between the health functions (e.g. financing and regulation) and actors involved (i.e. public, professional, private), and their impact in care delivery and outcomes. The purpose of this study is to analyse the interactions between PHC functions and their impact in PHC delivery, particularly in providers’ behaviour and practice organisation.

**Methods:**

Following a systems thinking approach with data obtained through a three-round European Delphi process, we developed a framework that captures (1) the interactions between PHC functions by analysing correlations between PHC characteristics of participating countries, (2) how actors involved shaped these interactions by identifying the actor and level of devolution (or fragmentation) in the analysis, and (3) their potential effect on care delivery by exploring panellists’ opinions.

**Results:**

A total of 59 panellists from 24 countries participated in the first round and 76% of the initial panellists (22 countries) completed the last round. Findings show correlations between governance, financing and regulation based on their degree of decentralisation. This is supported by panellists, who agreed that the actors involved in health system governance determine the type of PHC financing (e.g. ownership or payment mechanisms) and regulation (e.g. competences or gatekeeping), and this may impact care delivery and outcomes. Governance in our framework is an overarching function whose impact in PHC delivery is mediated through the degree of decentralisation (both delegation and devolution) of PHC financing and regulation.

**Conclusions:**

The application of this approach in policy implementation assessment intends to uncover limitations due to poor accountability and commitment to shared objectives. Its application in the design of health strategies helps foresee (and prevent) undesired or unexpected effects of narrow interventions. This approach will assist in the development of the realistic and long-term policies required for health systems strengthening.

**Electronic supplementary material:**

The online version of this article (10.1186/s12961-019-0456-8) contains supplementary material, which is available to authorized users.

## Background

The ‘Health for All’ movement started in the 1970s and set the path towards the current goal of universal health coverage. Its principles were assembled in 1978 in the Declaration of Alma-Ata, which called for a political commitment to implement sustainable and integrated primary health care (PHC) as the essential care for all the individuals in their communities [1]. The international endorsement of the declaration anticipated a turning point in the organisation of health systems towards health promotion and disease prevention, and a multisectoral action to tackle socioeconomic determinants of health. However, the political and economic instability of the succeeding years hindered its implementation. In the European area that concerns our study, the fall of the Soviet Union left many countries in transition in a global environment of market-driven reforms and constrained budget allocation to public services [2–4]. Worldwide, the spread of HIV/AIDS, tuberculosis and malaria contributed to the loss of advocates for holistic PHC in benefit of selective approaches [5]. Selective PHC proposed more objective and accountable targets to allocate health resources and, thus, attracted the political and economic efforts required to pursue the Alma-Ata commitments [6]. This was also supported by the World Bank and international donors, which played an important role in health care agenda-setting at that time [7, 8]. The holistic PHC approach itself was also commonly misunderstood [9] – it was either considered as inexpensive health care only appropriate for rural areas and developing countries or as unaffordable and utopic. It was also criticised for being focused on people’s assumed health needs instead of looking at health demands [10].

However, the last decades witnessed a surge in health care demands due to aging populations and multimorbidity [11], entailing a threat to health systems structured around specific diseases and driving the political and public interest towards health care integration and comprehensive PHC once again [12–14]. WHO called for a reintroduction of PHC in the global health agenda in 2008 [15]. Thus, efforts to strengthen PHC have continued and increased during the last decade, with a renewal of the stakeholders’ commitment to its implementation in the 2018 Global Conference on Primary Health Care in Astana, Kazakhstan [16]. An integrated and holistic PHC is a valuable resource for health promotion and prevention and management of diseases in both developed and developing countries [17], and an ally in the achievement of universal health coverage and sustainable development goals [18].

PHC systems have been previously described according to Donabedian’s structure–process–outcome triad [19] and Starfield’s PHC core attributes of accessibility, comprehensiveness, coordination and continuity [20]. The PHC structure has been defined as the group of logistic, financial, human and infrastructural resources that enables the process of health care delivery [19]. The process can be understood as the act of providing and receiving health care itself and is measured in its capacity (e.g. facilities), outputs (e.g. interventions), and impact on intermediate (e.g. indicators of disease management) and immediate outcomes (e.g. health status, equality, costs) [21–23].

Over the last 40 years, efforts to restructure and decentralise health systems have emphasised the importance of regulation and accountability in the multi-stakeholder arena and caused growing interest in PHC and health systems governance [24–26]. The balance of actors involved (i.e. public, professional and private) in health system functions has been an important criterion for health systems classification and analysis of European and OECD health systems [27–29]. In contrast, this has not gained the same attention in the analysis of PHC [22, 30, 31]. In multi-stakeholder health systems, it is difficult to pursue a shared objective fuelled by the sense of belonging or affiliation to the same system. The importance of considering the actors involved lies in the governing capabilities that this involvement confers and the potential conflicts of interest in the development of their functions [25, 32, 33].

Moreover, research in PHC performance has often focused on the impact that individual interventions (e.g. payment mechanisms) have in care delivery and has not considered the potential interactions between different functions (e.g. financing and regulation) in their joint effort to deliver health care [22, 31]. Health system functions, such as resource generation and service delivery, are not performed in isolation and, thus, should not be analysed independently. The analysis of PHC with a systems thinking approach considers the dynamism between functions and actors in their synergistic effort of health care production [34–37]. The aim of this study was to analyse these interactions and how they may impact PHC delivery, particularly professionals’ use of resources and practice organisation, as well as health outcomes. To achieve this, we conducted a multi-country Delphi process, the results of which are integrated with a literature review to define a PHC framework that captures these interactions.

## Methods

### Study design

We follow a systems thinking approach, which allows a wide perspective including different functions, actors and their interrelations [34, 38]. A pillar of systems thinking analysis is accounting for ‘who does what’ when analysing the systems’ functioning. In theory, this will help explain ‘how it is done’, based on the synergies and dynamism between all system elements [34]. In our study, this pillar is captured by describing each PHC function in terms of (1) ‘who does what’ – the actor involved (i.e. public, professional, private) and degree of devolution (e.g. central, regional) or fragmentation (e.g. one or several bodies), and (2) ‘how is it done’ – the mechanism employed to fulfil the function (e.g. type of payment, type of employment status). This can be considered as a result of or cause for the type of actor involved (system-as-cause thinking) [34].

This was analysed with a mixed-methods design that combined a literature review with quantitative and qualitative analyses of data obtained through a three-round multi-country Delphi process. This allowed the redefinition of an initial conceptual framework (informed by the literature) with inputs obtained through empirical analysis of survey data and additional evidence found in the literature.

The Delphi process is a group facilitation technique, developed by the RAND corporation, that uses iterative questionnaires to explore the opinions of a panel of experts or achieve consensus on a particular topic [39]. Our panel consisted of PHC academicians and physicians with experience in PHC service research and based in the WHO European Region countries, who were purposively contacted following the WONCA (World Organization of Family Doctors) Europe Branch members list [40] and extended through snowball sampling. This regional limitation allowed for inclusion of different health system types while facilitating availability of comparable statistical data. We contacted panellists by email, provided the study information sheet and requested their signed informed consent for participation.

### Framework development process

**Fig. 1:**
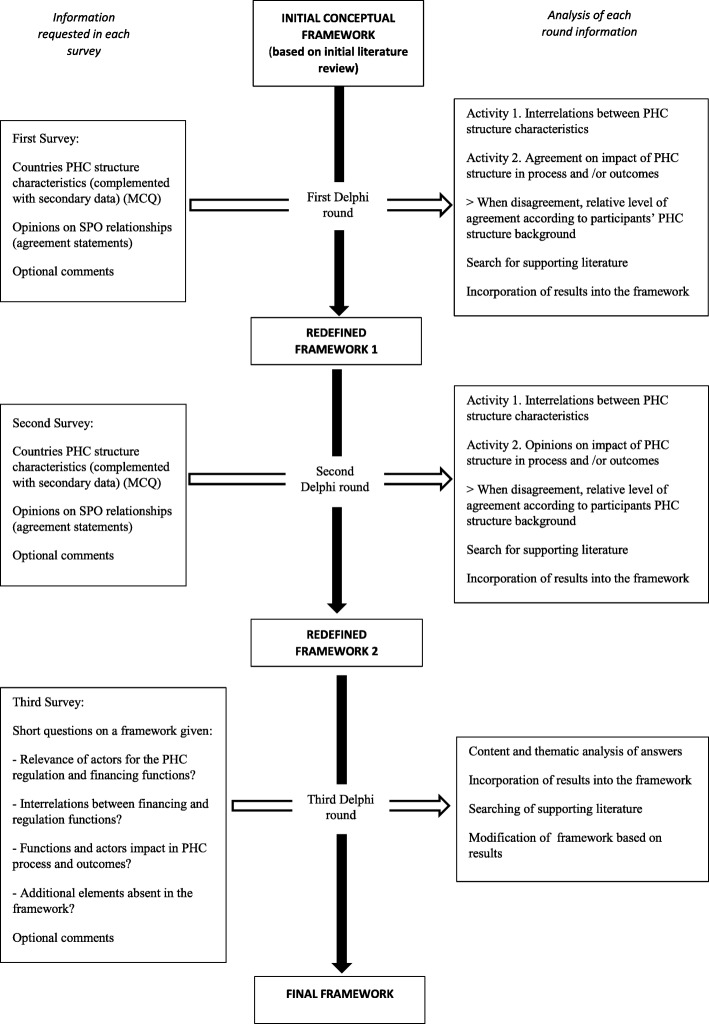
Study methods flow chart. *MCQ* multiple choice question, *PHC* primary health care, *SPO* structure–process–outcome

Figure 1 shows the framework development process. The literature review and Delphi rounds ran from February 2016 to May 2017. This included the time for initial review, panellist recruitment, survey development, pilot, response collection, data analysis, and feedback reports after the Delphi rounds.

The surveys were accessible online by invitation using the password-locked Qualtrics service (Salt Lake City, United States of America). The first survey was piloted in four of the participating countries (United Kingdom, Ireland, Germany and Spain), selected for having different PHC characteristics, in order to check the appropriateness and validity of the content in different health system contexts.

As depicted in Fig. 1, the redefinition of the initial framework over the first and second rounds was based on two main activities, as described below.

Activity 1. Quantitatively examining the interrelations between PHC functions (i.e. financing and regulation). We obtained information on Delphi countries’ PHC systems, which was recorded as the following categorical and binary variables informed by the literature (e.g. Sibthorpe and Gardner [41], Siddiqi et al. [42], Kringos et al. [21], Mikkelsen-Lopez et al. [25], and Schäfer et al. [43]):

1. Supply-side: health system financing; providers’ employment status and payments; facilities’ ownership; regulation of providers’ competences, clinical practice and license conferral; guidelines; and compulsory vocational training.
2. Demand-side: entitlements for receiving PHC, co-payments, gatekeeping.

Activity 2. Exploring the potential impact of these PHC functions in care delivery and outcomes. We assessed panellist agreement with literature-based statements on PHC structure, process and outcome [19] relationships using five-point Likert-scale questions.

The results of each round guided additional review in search for evidence that supported the redefinition of the framework. As depicted in Fig. 1, the final framework was obtained after the third-round survey, which directly asked panellists’ opinions on the functions and interrelations shown in the framework. Additional file 1 contains a list of variables and categories used in Activity 1 and Activity 2. Additional file 4 contains the statements used in Activity 2.

### Search strategy

We designed broad and narrow search strategies to capture studies on health systems performance and indicators, and evidence on how the PHC functions interact and impact delivery and outcomes. These combined keywords and MeSH terms were designed for Medline and adapted to EMBASE, Global Health and Health Management Information Consortium databases, and were limited to observational and review studies without language restrictions. We used titles and abstracts to identify relevant articles and checked their reference lists. We also reviewed reports published by international organisations (e.g. WHO, World Bank).

### Statistical analysis

We used IBM SPSS v24 for data analysis. For the description of the PHC characteristics of the Delphi panel countries, we identified the predominant category (e.g. main type of provider payment mechanism) and the range of categories present in the country (e.g. all types of provider payment mechanisms in use). When there were several panellists from a country, we used the mode of panellist responses. We contrasted the data collected with the information available in the Health Systems and Policy Monitor, developed by the European Observatory on Health Systems and Policy [44].

We calculated bivariate correlation coefficients (Pearson, Fisher’s test and likelihood ratio, alpha significance ≤ 0.05) to identify interrelations between any given two PHC characteristics (Fig. 1, Activity 1). Significant interrelations that were also supported by the literature constituted the basic functional network of our framework. Missing data was addressed via pairwise deletion.

In the analysis of five-point Likert-scale questions, we determined panel consensus when over 70% of the panel agreed on a structure–process–outcome statement (accepted degree of consensus in reviewed literature) (Fig. 1, Activity 2) [45]. When there were discrepancies, we analysed them against the panellist’s country PHC system (i.e. correlating the level of agreement with panel PHC information). This provided additional insight on the impact of national contexts in the panel’s level of agreement while intending to control for self-selection bias. Additionally, thematic analysis of panellists’ comments triangulated with the literature also contributed to establishing the functional interrelations and potential impact in care delivery and outcomes.

## Results

The process of development of the framework over the three Delphi rounds is provided in the supplementary materials (Additional file 2). In this section, we describe the final framework, obtained after the final round, and a summary of the supporting findings, obtained during the first and second rounds. This is preceded by a description of the Delphi panel and countries.

### Delphi panel description

**Fig. 2:**
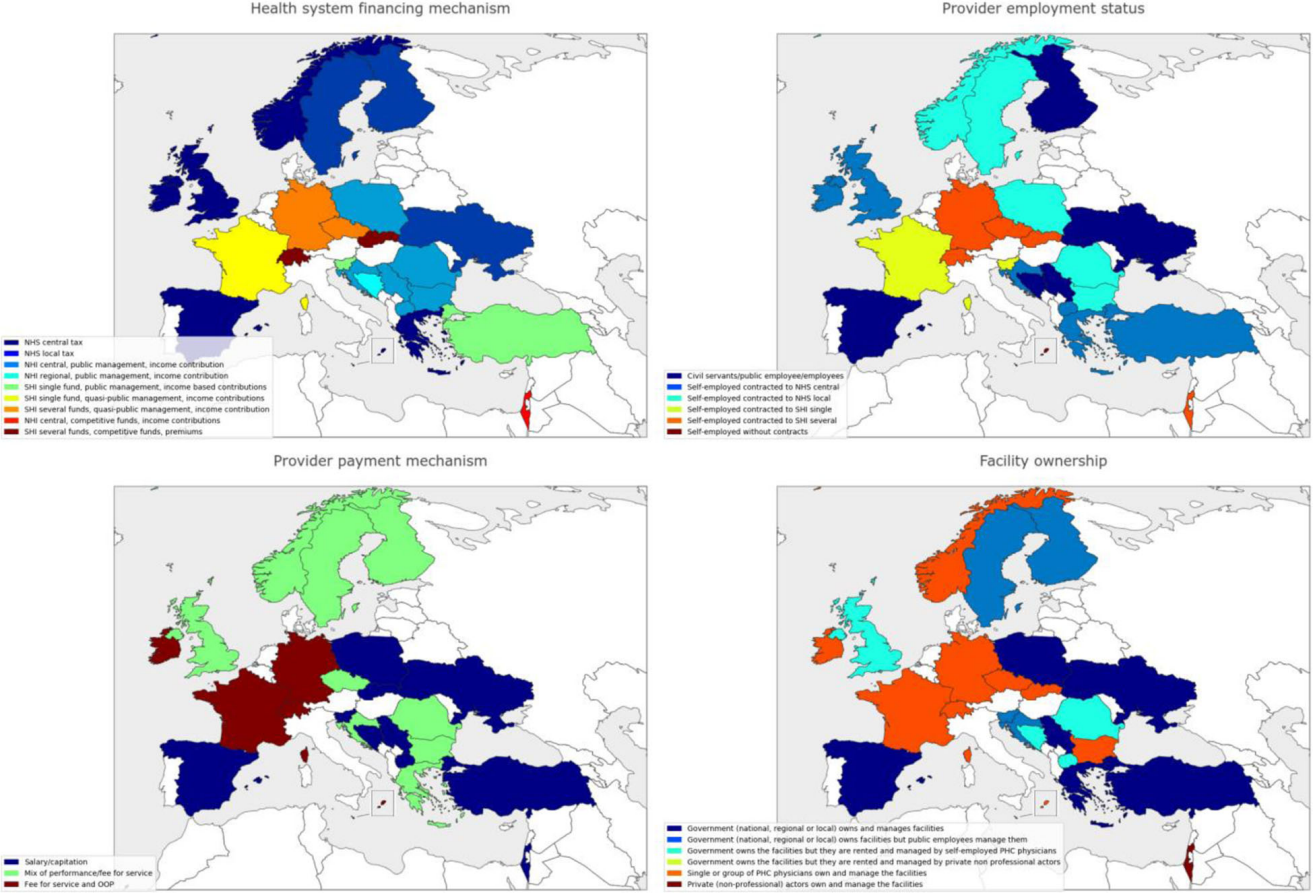
Selected health systems and primary health care (PHC) characteristics of Delphi panel countries. When several types of a health system or PHC characteristic are present in a country, the predominant category type is shown. NHS national health service, NHI national health insurance, SHI social health insurance, OOP out of pocket, PHC primary healthcare

**Fig. 3:**
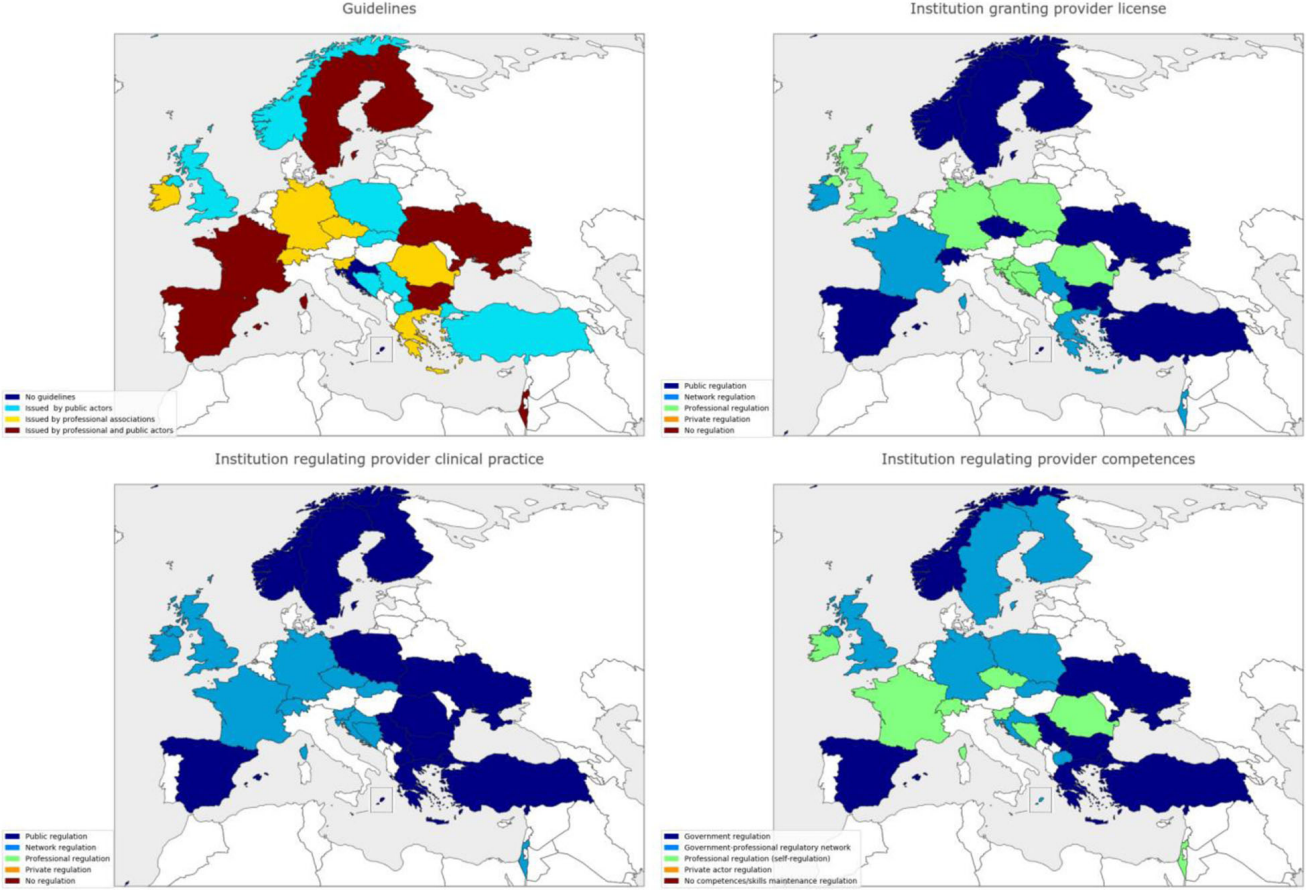
Selected health systems and primary health care (PHC) characteristics of Delphi panel countries. When several types of a health system or PHC characteristic are present in a country, the predominant category type is shown. NHS national health service, NHI national health insurance, SHI social health insurance, OOP out of pocket, PHC primary healthcare

**Fig. 4:**
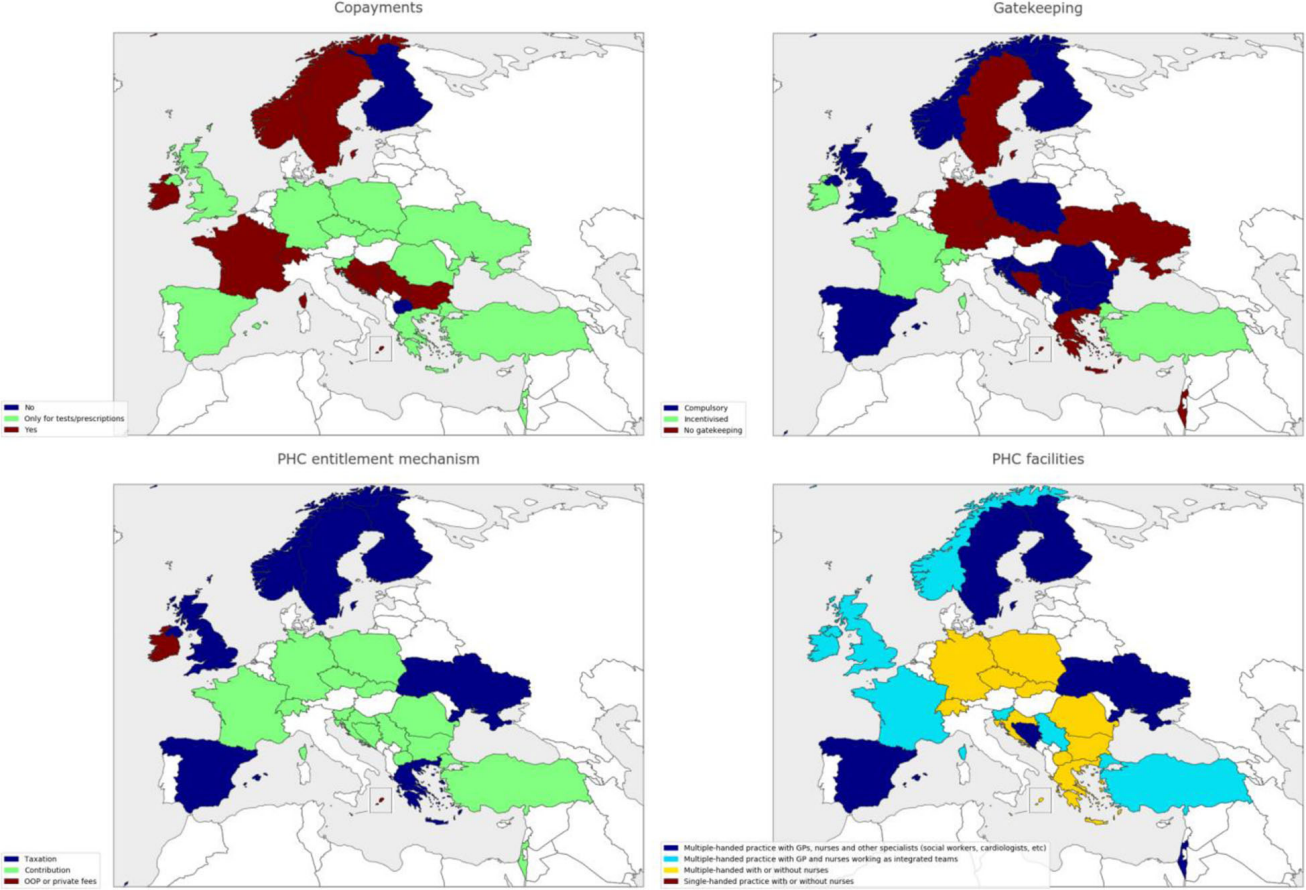
Selected health systems and primary health care (PHC) characteristics of Delphi panel countries. When several types of a health system or PHC characteristic are present in a country, the predominant category type is shown. NHS national health service, NHI national health insurance, SHI social health insurance, OOP out of pocket, PHC primary healthcare

Table 1 shows demographic and professional information of the Delphi panel. Out of 105 invitees, 70 accepted to participate and the actual participation in the first, second and third rounds was 59, 54 and 45 panellists, respectively (attrition rate of 23.5%). Regarding countries’ representation, 24 WHO European region countries, namely Bosnia and Herzegovina, Bulgaria, Croatia, Czech Republic, Finland, France, Germany, Greece, Ireland, Israel, North Macedonia, Malta, Norway, Poland, Romania, Serbia, Slovakia, Slovenia, Spain, Sweden, Switzerland, Turkey, Ukraine, and United Kingdom, were represented in the first and second rounds and 22 countries (the initial panel except Bulgaria and Slovenia) completed the final round. Figures 2, 3 and 4 depicts some of the characteristics of the countries’ PHC systems.

**Table 1:** Delphi panel description

Information	Round 1	Round 2	Round 3
Participation	59	54	45
Age			
< 35	5 (8.5%)	5 (9.4%)	5 (11%)
35–50	19 (32%)	16 (30%)	13 (29%)
51–70	34 (59%)	33 (61%)	27 (60%)
Current position(s) (panellists may hold more than one position)			
Primary health care physician	41 (71%)	38 (70%)	30 (67%)
Primary health care researcher/academician	44 (75%)	40 (74%)	32 (71%)
Professional association representative	22 (37%)	21 (39%	14 (31%)
Family medicine training body representative	17 (29%)	17 (31%)	14 (31%)
Health care manager/director	4 (5.8%)	4 (7.4%)	4 (8.9%)
Academic background			
Master’s degree	19 (32%)	17 (31%)	13 (29%)
PhD degree	28 (48%)	26 (48%)	19 (42%)
Medical specialisation in Family Medicine or other			
2–4 years in length	33 (56%)	30 (57%)	26 (58%)
> 4 years in length	13 (22%)	13 (24%)	9 (20%)

### Development of the framework

The initial literature review pointed out the relevance of financing, regulation and provision functions and type of actors involved (i.e. public, professional, private) for health systems analysis [27–29]. The initial framework included three broad functional blocks, namely (1) health system governance, characterised by the main health system financing mechanism, since it is informative on health system governing actors (i.e. relative role of state in governance) [46–48], (2) PHC financing of provision (staff and facilities) and reception (entitlements) of care, and (3) PHC regulation of provision (staff and facilities) and reception (demand) of care.

#### Framework description

**Fig. 5:**
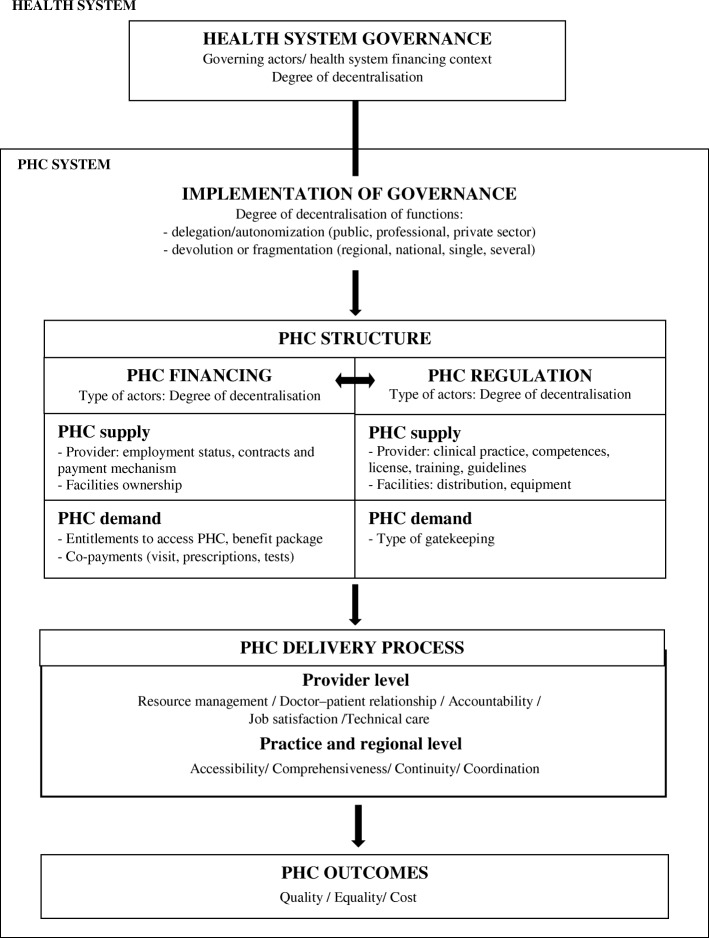
Primary health care (PHC) final framework

The final framework (Fig. 5) was obtained after the analysis of the third Delphi round. A total of 97% of the panel agreed that governance function (e.g. goals, policy-making, and definition of financing and regulatory mechanisms) impacts PHC delivery, particularly at practice level, but also at provider level [32, 49]. Additionally, 89% of the panel also agreed that these governing functions would be shaped by the balance of actors involved in their development “*as the shadow reflects the body*” (panellist’s comment) [27, 50].

Moreover, as depicted in Fig. 5, the results suggest that PHC financing and regulation are interrelated (e.g. type of regulation of providers depends on how and to whom they are contracted, or regulation of facilities distribution and equipment depends on the type of facilities ownership) [24]. Particularly, 89% of the panellists agreed that provider payment mechanisms have a role in the regulation of provider performance. This is based on an alignment between the actor responsible for financing (e.g. insurer or public institution contracting providers or developing infrastructure) and for regulation (e.g. professional or public institution in charge of training and competences development), who ultimately, as panellists agreed, influence how these functions are implemented [24, 32, 51]. Overall, 80% of the panel agreed that this could impact PHC delivery processes at provider level (i.e. technical care, resource management, accountability, job satisfaction and doctor–patient relationships) [52] and PHC practices at regional level (i.e. Starfield’s core attributes of accessibility, comprehensiveness, continuity, and coordination) [53], and ultimately impact outcomes, particularly health care quality, equality and costs (identified in Kringos et al. [21]) (Fig. 5).

As panellists pointed out and the literature supports [54, 55], quantifying the type of actor responsible for governance, financing and regulation is equivalent to quantifying their degree of decentralisation (both delegation – or autonomisation – and devolution) of the functions. The degree of decentralisation has been considered an indicator of health care governance [21, 42]. Then, as illustrated in Fig. 5, governance can be understood as an overarching function [25, 34, 47], whose impact in PHC delivery is mediated through the degree of decentralisation of financing and regulation [51]. Hence, PHC financing and regulation are secondary functions identified as ‘Implementation of Governance’ in our framework, as 93% of the panel agreed.

#### Supporting findings

The final framework was supported by first and second Delphi round findings. When examining the interrelations between functions and actors (Fig. 1, Activity 1), governance (i.e. health system financing mechanism used as proxy of governing actors, as indicated before) was correlated to both PHC regulation, such as type of institution regulating physicians’ clinical practice (Pearson correlation (PC) = 0.519, *p* = 0.009) and conferring the license (PC = 0.493, *p* = 0.014), and PHC financing such as physicians’ employment status (PC = 0.410, *p* = 0.047) and facilities’ ownership (PC = 0.592, *p* = 0.002). For example, in our Delphi panel, the national health service type was correlated to having public employee physicians (PC = 0.410, *p* = 0.047), government ownership of facilities (PC = 0.655, *p* = 0.001), having physician competence and licenses regulated by public institutions (PC = 0.434, *p* = 0.034 and PC = 0.480, *p* = 0.018, respectively), and clinical practice regulated by employer institutions (PC = 0.607, *p* = 0.002). Social health insurance (single or several funds) types were correlated to having self-employed physicians (PC = 0.434, *p* = 0.034), fee for service (FFS) payments (PC = 0.747, *p* = 0.000), and physicians’ clinical practice regulated by a professional college (PC = 0.513, *p* = 0.010). The financing and regulatory functions have been previously included as governing mechanisms [42, 46, 47]; for example, Sidiqi et al. [42] identified contractual arrangements and incentives, regulation of physicians and the role of the Ministry of Health in these as governance indicators.

As depicted in Fig. 5, PHC financing and regulation were also interrelated. For example, facility ownership (i.e. a PHC financing indicator) correlated to the type of institution regulating physicians’ competences and clinical practice (i.e. PHC regulation indicators) (PC = 0.481, *p* = 0.017 and PC 0.418, *p* = 0.042, respectively). Actually, Crampton and Starfield [53] identified the governing and regulatory capabilities attributed to facility ownership, as well as Hsiao [56], who also pointed out that ownership can “*determine to whom and for what an organisation is held accountable*”. In this way, government ownership correlated to public institution regulating physicians’ clinical practice and licenses (PC = 0.540, *p* = 0.006 and PC = 0.467, *p* =0.022, respectively), or private non-professional ownership correlated to lack of clinical practice regulation of physicians (PC = 0.692, *p* = 0.000) in our panel (see Additional file 3 for correlations table with additional results).

When exploring the potential impact of financing and regulatory functions in care delivery and outcomes (Fig. 1, Activity 2), we classified the statements according to the function they referred to (i.e. governance, financing or regulation), and subclassified them according to the delivery attributes (i.e. access, coordination, comprehensiveness or continuity) and outcomes alluded to in the statements (Table 2).

**Table 2:** Agreement with functions (financing, regulation, governance) during the Delphi process

Functions	First round statements agreement	Second round statements agreement	Third round questions agreement
Financing	7statements	8 statements	12 short questions
Agreement (%)			
- ≥70%	1/7	3/8	9/12
- 65-70%	2/7	1/8	3/12
- 60-65%	1/7	0/8	0/12
Regulation	7 statements	5 statements	10 short questions
Agreement (%)			
- ≥70%	2/7	5/5	10/10
- 65-70%	0/7	0/5	0/10
- 60-65%	0/7	1/5	0/10
Governance (actors)	4 statements	5 statements	12 short questions
Agreement (%)			
- ≥ 70%	0/4	2/5	12/12
- 65-70%	3/4	0/5	0/12
- 60-65%	0/4	0/5	0/12
Overall agreement	18 statements	18 statements	34 short questions
- ≥ 70%	3/18	10/18	31/34
- 65-70%	5/18	1/18	3/34

Table 3 shows examples of the statements’ analysis (Additional file 4 contains all statements). PHC financing may affect providers’ job satisfaction and impact quality. For example, 91% of the panellists agreed that self-employed physicians (i.e. type of employment status included in PHC financing) are attributed additional management and bureaucratic work compared to public physicians (Table 3, statement one), leading to burn-out. This links PHC financing (i.e. employment status) with physician workload, which may impact job satisfaction and quality of care [30]. According to panellists, this contributes to the low take-up of family medicine/general practice residence among medical students. Professional behaviour can also be affected. For example, 75% of the panel agreed on the influence that patient entitlements (i.e. a demand-side PHC financing indicator) may have on physician behaviour and lead to an unjustified increase in access to services and health care costs (Table 3, statement two). Panellist comments pointed out the importance of physicians’ competences and ethics, on the one hand, and of doctor–patient communication, on the other, in mitigating this influence. This is supported by the correlations between patient entitlements and physician regulation identified with Activity 1 (Fig. 1).

**Table 3:** The impact of governance in primary health care delivery: a systems thinking approach with a European panel

Statement examples	Correlation between agreement /disagreement and panel PHC background	Thematic analysis (aspect of structure-process-outcome referred in the statement)
1. Setting up and managing PHC practices constitute additional workload for self-employed PHC physicians, compared with public employee physicians. A: 91% N: 5% D: 4%	A: SHI financing mechanism, self-employed physicians, compulsory training.	Structure: PHC financing; Process: Job satisfaction; Outcomes: Quality
	**Comment example:** “Practice management is a job. In France, new practices trying to put preventive care forward struggle because of the additional workload (e.g., asking for funds, meeting representatives, motivating other practitioners toward new health .care practices, etc)” (agree)	
	**Comment example:** “Public PHC centres have administrative staff and director, in private PHC there is none and one GP makes this part of the job (or it is externalised, which increases costs)” (agree)	
2. Private patients' expectations for diagnostic and treatment activities can be high as they feel they are contributing more to the care they receive, and this may lead to unnecessary interventions or treatments. A: 75% N: 13% D: 12%	A: Private ownership, lack of NHS contracts.	Structure: Patients’ entitlements (employment status, payments); Process: Access; Outcome: Costs
	Level of agreement: OOP (no significant differences between subgroups)	
	**Comment example:** "When patients go to doctors privately, it is almost as if the doctor has to give the patients their "money's worth" (agree)	
	**Comment example:** “It depends on the degree of patient’s relationship with their physician and their educational level” (disagree)	
3. When physicians are monitored on their clinical practice, the same organisation that monitors them should provide clinical guidelines to support them (in order to ensure some consistency between the guidelines and the clinical practice monitored!). A: 71% N:10% D: 19%	A: PHI or OOP coverage, competence regulated, physicians’ competences regulated by central or regional.	Structure: PHC regulation; Process: Accountability, Compliance; Outcomes: Quality
	D: General taxation entitlements, lack of OOP or PHI entitlements, lack of competences regulation	
	**Comment example:** “However, the guidelines must be done with physicians’ approval or it will lead to a direct conflict…” (agree)	
	**Comment example:** “They should count on national guidelines” (disagree)	
4. Public planning of the distribution of PHC services can help decrease the inequalities in access to PHC in a country. A: 89% N: 9% D: 2%	A: Civil servants, lack of FFS payments, type of institution conferring the license to practice.	Structure: PHC regulation; Process: Access; Outcomes: Equality
	**Comment example:** “Self-organisation leads to less and less GPs working in deprived and poor area. Public health centres allowed to increase the number of GPs in those areas” (agree)	
	**Comment example:** “We must pay attention to freedom of exercise in case of public planning” (disagree)	
5. The coordination of PHC physicians with other specialists/hospital services can be difficult. Health authorities should establish clear links and pathways to make this coordination easier. A: 87% N: 5% D: 8%	A: Contracted to NHS or NHI, capitation and performance payment mixed.	Structure: PHC regulation; Process: Coordination; Outcomes: Quality
	D: SHI several funds financing, FFS payments	
	**Comment example:** “Clear and easy to follow” (agree)	
	**Comment example:** “This will generally increase the bureaucratic workload” (disagree)	

A: Agreement, N: Neither agreement nor disagreement, D: Disagreement

Potential misalignment of actors should be considered when implementing regulation and exercising accountability [30, 52]. In statement three, 71% of the panel agreed that the institution holding physicians accountable for their clinical practice should be the same with the institution developing guidelines to avoid inconsistency between the practices regulated and recommended. This ultimately may lead to an increase in care quality [32] (Table 3, statement three). Regarding practice level organisation, 89% of the panel agreed on the role of the state in decreasing inequalities in access to services [57], which links PHC regulation to accessibility and equality of care delivery (Table 3, statement four). Similarly, 87% of the panel agreed on the role of the state in facilitating the coordination between health services, which may impact the quality of care (Table 3, statement five).

**Fig. 6:**
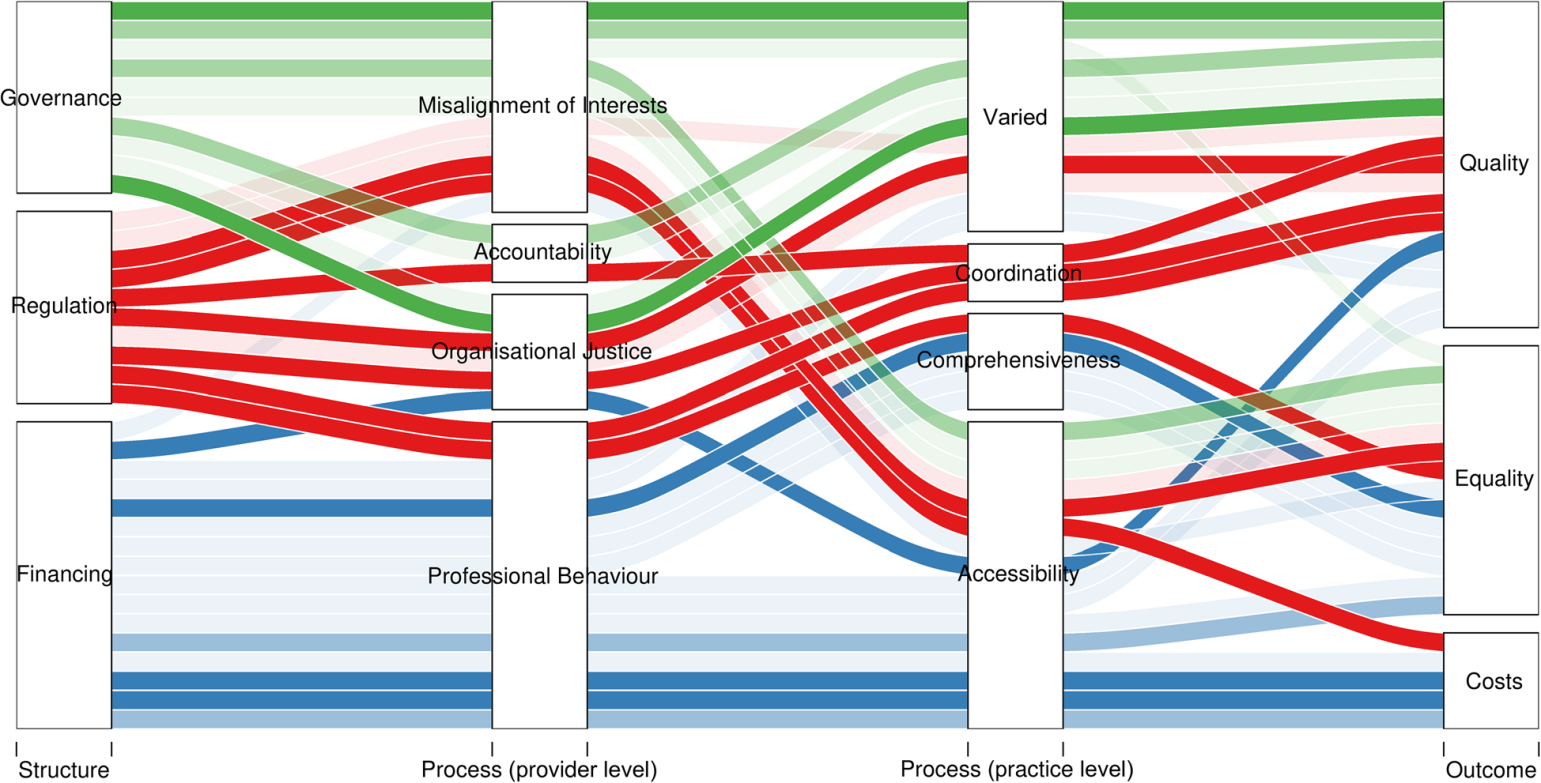
Alluvial diagram depicting the level of agreement with structure–process–outcome statements. In the analysis, statements are classified according to the structural function referred to (i.e. governance/governing actors, regulation, financing). Subsequently, statements are classified according to the provider’s and practice’s attributes potentially influenced by the structural function. Following this, statements are classified according to the outcome attribute alluded to. Information is obtained through thematic analysis of the statements. Strongest colour depicts agreement over 70%, medium strength colour depicts agreement between 65% and 70%, minimum strength colour depicts agreement lower than 65%.

In general, as depicted in Fig. 6, panellists agreed that PHC financing may impact care costs by influencing service accessibility, and impact equality through its influence in comprehensiveness and accessibility of PHC [58]. PHC regulation may impact care quality by influencing services coordination, and equality by influencing PHC accessibility and comprehensiveness [59]. Governing actors may influence care quality via shared goals, accountability and organisational justice processes, and equality by influencing accessibility of PHC [58].

To illustrate our approach, Table 4 compares a possible national strategy to increase the PHC role in diabetes management in two countries – Spain, which has a public devolved health system, and Slovakia, which has a compulsory competitive insurance model [44]. In Spain, policy dialogue and planning have the central and regional Ministries of Health (MoH) as main actors, advised by the professional organisations and patient groups (Table 4). Since there is no purchaser–provider split, financing mechanisms for the implementation by the regional MoH would involve refinement of providers’ contract and incentives and, as the owner of PHC facilities, adjustment of equipment and skill-mix for diabetes management. Regulatory mechanisms, also by the regional MoH, would be encompassed within providers’ contracts. The general MoH supervises and provides clinical guidelines jointly with professional organisations (Table 4) [44]. In Slovakia, policy dialogue and planning would involve MoH, health insurance companies (HICs), the health care surveillance authority (HCSA) and medical chambers [44]. Financing mechanisms for the implementation would require HICs to adjust providers’ contracts and incentives. Providers, as owners of facilities, would be responsible for adequate equipment and skill-mix. The implementation would be supervised by the HCSA and self-governing regions (SGRs), the latter in charge of providing the permits to HICs, practices and providers (Table 4) [44]. Regulatory mechanisms would be implemented by the HCSA and SGRs, who supervise the quality of providers and facilities, along with the medical chambers, who regulate providers’ competences. The HICs supervise the providers’ clinical practice, which is informed by MoH clinical guidelines (Table 4) [44].

**Table 4:** Actors and mechanisms required to implement a national strategy to increase PHC role in diabetes management

	Spain	Slovakia
Policy dialogue, situation analysis, planning	- Actors: MoH (central and ACs), providers and patient groups (advisors) MoEc approves budget and extra funds to support ACs - Regional MoH: adapts policy/plan to ACs	- Actors: MoH, HICs, health care provider representatives, HCSA
Implementation PHC Financing	- Regional MoH: inclusion of activities for diabetes management in providers’ contracts, incentives (P4P) or non-financial incentives, provider/practice objectives, accessibility to PHC and availability of equipment for diagnosis/management at PHC level, skill-mix or multidisciplinary practices, integrated electronic records - Central MoH: earmarked funds to implement strategy (if necessary)	- HICs: inclusion of activities for diabetes management in providers’ contracts, payment alignment (P4P or FFS), provider/practice objectives, availability of equipment for diagnosis/management at PHC level - SGRs: accessibility of diagnosis and management services, ensures minimum access via facilities ownership (mostly hospitals)
Implementation PHC Regulation	- Regional MoH: supervises competences and monitors achievement of management objectives (pre-specified and aligned to MoH guidelines) - MoH: provides clinical guidelines, supervises implementation is aligned with national strategy - MoE: provides license - Professional organisations (SEMFyC and regional branches) collaborate with MoH for guidelines development	- HCSA: supervises MoH, HICs, SGRs, providers - Medical chambers: regulate competences provide license (membership not compulsory) - SGRs: provide permits to HICs, providers and facilities - MoH: provides clinical guidelines, develops quality indicators - HICs: regulate diabetes management, measure quality indicators
Resistance/challenge	- PHC postgraduate training curriculum’s adaptation to enhanced scope of practice (MoH, MoE and professional associations) - Providers’ inclusion in policy dialogue and planning alignment of payments/incentives across health services, and development of care pathways could enhance coordination of diabetes management and acceptance of PHC role - Budget constraints and competitions for public funds may limit access to diagnostic services in PHC services - Uneven implementation of national strategy in ACs: central support and additional earmarked funds could aid - Budget miscalculation for implementation and maintenance of strategy may lead to unsustainable/temporary reforms - Untargeted conditions (diseases not covered under specific disease programme) may be neglected – supportive guidelines, comprehensive PHC physicians training and continuous education may be helpful	- PHC postgraduate training curriculum’s adaptation to enhanced scope of practice (MoH, MoE and Medical Chambers) - HICs should incentivise group practices - Secondary/inpatient care may resist gatekeeping – inclusive policy dialogue and payments/incentives alignment across health services may dissipate resistance - Population resistance due to reduction of freedom of choice – population trust for the services through raising awareness campaign may dissipate resistance - Distribution of providers, diagnostic and therapeutic services for diabetes management may vary across country - Patients/civil society representation – inclusion may improve population awareness/acceptance of PHC - Untargeted conditions (diseases not covered under specific disease programme) may be neglected – supportive guidelines, comprehensive PHC physicians training and continuous education may be helpful
Sector-wide approach opportunities	- Public health programmes to tackle risk factors and encourage healthy lifestyle – inclusion in policy dialogue and planning - Cross-sectorial collaboration (Health in All policies) – food, transport	- Public health programmes to tackle risk factors and encourage healthy lifestyle – inclusion in policy dialogue and planning - Cross-sectorial collaboration (Health in All policies) – food, transport

*ACs* autonomous communities, *FFS* fee for service, *HCSA* Health Care Surveillance Authority, *HICs* health insurance companies, *MoE* Ministry of Education, *MoEc* Ministry of Economy, *MoH* Ministry of Health, *P4P* pay for performance, *SEMFyC* Spanish Family and Community Medicine Society, *SGRs* self-governing regions

## Discussion

We have applied a systems thinking approach to analyse the impact of PHC structure in delivery, particularly in providers’ use of resources and practice organisation, and outcomes, which were gaps identified in the literature [23]. Governance in our framework would impact PHC delivery through the secondary functions of PHC financing and regulation, whereas the level of decentralisation of these determines the way in which health system governance is implemented [55]. As panellists agreed, the degree of decentralisation may determine the policy-making process (e.g. share of public–private actors may affect health system’s goals and objectives, including the relevance of equity among them) [50], and influence care delivery through types of contracts and ownership (‘hard governance’) or regulatory and payment mechanisms (‘soft governance’) [48, 49]. This can affect physicians’ management of resources, job satisfaction and accountability, and influence the accessibility (e.g. facilities and providers’ distribution and co-payments), coordination and comprehensiveness (skill-mix, equipment) of the PHC provided [50].

Applying a systems thinking approach when designing policies or evaluating specific interventions helps foresee (and prevent) or interpret the effects of these interventions at levels different to those initially targeted [35, 36, 57]. For example, resistance to the diabetes strategy in Slovakia could be higher than in Spain since secondary care physicians are also independent providers, with FFS income, and could oppose its implementation or any type of gatekeeping due to competing interests (Table 4). In Spain, most of secondary and tertiary care is public and paid on a salary basis, which facilitates the working gatekeeping. Emphasising inclusive policy dialogue with secondary and tertiary care providers (in the Slovakia case but also important in Spain) could create a positive environment for developing shared goals and care coordination pathways, and promoting gatekeeping implementation (Table 4) [60].

In PHC performance analysis and comparison, our framework also intends to reveal limitations in health system functioning (e.g. in policy implementation or adherence to guidelines) drawn from poor communication, accountability or commitment to shared goals, and may inform interventions to improve local and regional health care management [26, 49, 59]. For example, providers’ adherence to the diabetes guidelines issued by the MoH in Slovakia would be advocated by the medical chambers and operationalised by HICs (Table 4) since these are the organisations to whom providers may feel more directly accountable. Likewise, PHC delivery can be also perturbed if the direct responsibility for care provision relies with professional or private actors, e.g. self-employed physicians made responsible to guarantee geographical access or affordable care. This may directly impact the distribution of facilities or provision of specific services with population and individuals’ health repercussions, and require policy action, even more when health care is financed with public resources. For example, SGRs in Slovakia are responsible for ensuring health care accessibility, even with direct provision, if HICs or providers leave gaps (Table 4).

In multiple-stakeholder health systems, it is difficult to pursue a shared objective fuelled by the sense of belonging or affiliation to the same system [50]. The degree of decentralisation can be understood as an indicator of alignment of objectives between the actors involved. However, it is important to mention that decentralisation of health systems is founded in different rationales. It may be based on the cultural background of the country, and then it would represent trust between health systems actors and recognition of their legitimacy to participate [26, 50, 54]. It may have a political rationale and attempt to represent different actors’ interests. Finally, it may seek to impact performance, aligned to other policies to increase responsiveness and efficiency of health systems. When assessing decentralisation processes, either the achievement of legitimacy, interest representation or performance may be used as indicators, and using the three of them is recommended [54].

Delphi methods are appropriate for model development; however, there are some recognised limitations [39]. The strength of the study partly depends on the strength of the panel. Panellists’ inclusion was based on their experience in PHC (e.g. clinicians, managers) and commitment to the study. This contributed to internal validity through panel heterogeneity and low attrition rate; however, their level of expertise on the topic of study may differ. This was addressed with definition of core concepts, informed feedback and repetition across rounds. Researcher bias may also arise from question selection and controlled feedback. This was addressed by building on an initial framework based on the literature as well as supporting the results with different methods and sources of evidence (i.e. panellists’ absolute and relative agreement with statements, qualitative analysis of comments and literature).

Additionally, the framework is tilted towards the supply side, focused on factors that may influence physicians’ use of resources and practice organisation and with only a few demand-side characteristics included. Other factors affecting PHC demand, such as socioeconomic determinants, are not represented. The same is true for other health systems organisational and financial factors influencing PHC delivery, such as secondary or tertiary care availability or expenditure. This was suggested by panellists and should be accounted for when applying the framework for performance analyses. Besides the supply-side focus, the relationships between functions are based on linear correlations, and this is not ideal for a systems thinking approach, for which non-linear or dynamic modelling techniques are more appropriate [34]. This was limited by the lack of quantitative indicators for the functions observed. Considering health systems as hybrids, the measurement of decentralisation in cross-country analyses should reflect not only the categories available (e.g. types of facilities’ ownership) but also their weight (e.g. percentage of public expenditure in facilities or FFS amount paid to providers per each insurer). This would provide a more comprehensive picture of the health systems; however, this information was not available for all participating countries. Finally, we point out that the discussed impact of governance, financing and regulation in care delivery and outcomes (Fig. 6) is based on panellist opinions and supportive literature. This is otherwise useful for generating hypotheses, but these must be tested in future studies.

The framework is currently being applied to produce a taxonomy of PHC systems (using information of 24 Delphi countries as starting point) based on different degrees of decentralisation of PHC financing and regulation. The next step is the comparison of the taxa on non-communicable disease outputs and outcomes. For single country studies, the framework can be applied to establish within-country differences in health outputs and outcomes as a function of the PHC characteristics analysed using lower level data. This would be especially relevant for countries such as Spain and the United Kingdom, where autonomous regions or devolved governments have significant involvement in the running of the health system. Given quantitative data is available, the framework can be used as the basis of a model (an untested example using Unified Modelling Language notations [61] is provided in supplementary materials, see Additional file 5) for performance analysis with more suitable methods. The refinement of the model after calibration with country data would also improve the external validity of the framework.

Regarding the generalisability of the findings, the relationships supporting this framework were identified using data from 24 WHO European region countries and with the purpose of developing a taxonomy. Due to this empirical approach, some health systems actors and mechanisms are not represented in this study (e.g. role of donors, non-governmental organisations or faith-based organisations in health care). The application of this framework to assess the implementation of health programmes or policy reforms at local, regional or national level in other countries is encouraged and would require previous identification of actors, financing and regulatory mechanisms to analyse the existence of the interrelations here identified. When considering only the actors here included, we anticipate that the framework may be better applied in contexts where the state is, to some extent, constitutionally or legislatively responsible for population health, and there exists a mainstream health system financing mechanism (with or without other identifiable financing mechanisms) in order to better detect actors and accountability relationships.

## Conclusions

Our framework introduces a systems thinking approach in the analysis and comparison of PHC systems by uncovering the relationships between governance, financing and regulation, which we identified on the basis of their degree of decentralisation. The framework, which was validated by the expert panel, is being applied to classify countries according to the degree of decentralisation of PHC financing and regulation and compare their performance. This will provide a measure of the impact of PHC governance in care delivery. Using the framework in health policy implementation or performance assessment intends to reveal limitations in the performance of providers or facilities due to poor accountability and commitment to shared objectives, and point out the areas for improvement of health care management at local, regional or national level [26, 62]. Recognition of the relationships between health systems functions and a priori identification of their degree of decentralisation (i.e. actors involved and level of devolution or fragmentation) provide valuable information on the arena in which policies are designed and implemented, and helps avoid shortcomings by ensuring proper integration of actors through inclusive policy dialogue and planning, and alignment of mechanisms across health systems functions [63]. Using this approach helps foresee the cross-sectoral impact of interventions and prevent their undesired or unexpected effects within and beyond health systems boundaries [50, 57, 64]. This will contribute to the development of the realistic and long-term policy solutions required for health systems strengthening and the attainment of the highest possible level of health for all once endorsed [1, 18, 34].

## Additional files

Additional file 1: List of variables and categories used in the study. (PDF 97 kb)

Additional file 2: Evolution of the framework through three Delphi rounds. (PDF 107 kb)

Additional file 3: Correlations between PHC characteristics of Delphi panel countries. (PDF 176 kb)

Additional file 4: Statements classified into governance/governing actors, financing and regulation functions. (PDF 160 Kb)

Additional file 5: Framework described using unified modelling language notations. (PDF 659 kb)

## Abbreviations

FFS: fee for service
HCSA: Health Care Surveillance Authority
HICs: health insurance companies
MoH: Ministry of Health
P4P: pay for performance
PC: Pearson correlation
PHC: primary health care
SGRs: self-governing regions
