# Supplementary material for: The impact of governance in primary health care delivery: a systems thinking approach with a European panel
Source: Health Res Policy Syst. 2019 Jul 4;17:65. doi: 10.1186/s12961-019-0456-8 (PMC6609383; doi:10.1186/s12961-019-0456-8)
Supplement: Supplementary file 1 — List of variables and categories used in the study. (PDF 97 kb) [file 12961_2019_456_MOESM1_ESM.pdf]

The impact of governance in primary health care delivery: a systems thinking approach with a European panel

Authors: Ana Belén Espinosa-González, Brendan C Delaney, Joachim Marti, Ara Darzi

Additional file 1. List of variables and categories used in the study

Table 1 List of PHC variables included

**PHC supply and demand side variables**	
**Round 1**	
**Variable**	**Categories**
Providers' employment status	Civil servant/public employee<br>Salaried or employed by other self-employed PHC physicians or PHC practice<br>Self-employed<br>Others
Type of contract when is self-employed	Contracted to the national health authority<br>Contracted to local or regional health authority<br>Contracted to social health insurance (SHI) (single fund)<br>Contracted to SHI funds<br>Contracted to one or several private health insurance(s) (PHI)<br>Self-employed without contracts<br>Others
Providers' type of payments	Fixed salary<br>Mix of payment per performance or results +- capitation /basic payments<br>Fee for service (FFS)<br>Directly paid out-of-pocket (OOP)<br>Others
Type of mix of payment when selected	Mainly capitation and additional payment per performance/results (which one?)<br>Mainly payment per performance/results and additional payment per capitation (which?)<br>Balanced mix of payment per performance/results and capitation (which?)
If fee for service, who pays the fee?	Paid by national health insurance fund<br>Paid by SHI fund<br>Paid by PHI<br>Others
Ownership	Free text
Compulsory training programme for GPs	No/ Yes (duration)
Institution that issues professional license to practice	Free text
Providers' clinical practice regulation	The organisation they work for<br>The central organisation in charge of purchasing PHC services centrally<br>The local or regional organisation delegated to pay PHC services in that region<br>An independent inspection authority<br>The same institution that collects funds to finance PHC services<br>There is no institution monitoring<br>Others
Providers' competence regulation	The college of GPs/physicians<br>Another professional body (which one)<br>The same institution providing the license to practice<br>Their employer/ institution they have a contract with<br>Independent governmental body<br>The central/local health authority/ Ministry of Health (Mohr)<br>There is no institution in charge of monitoring<br>Others
Providers' guidelines	Yes/ no<br>Professional associations<br>Mohs/regional health authority<br>Pharmaceutical bodies<br>The same institution they are contracted to<br>The institution that holds physicians accountable for their clinical practice<br>An independent authority<br>Others
Gatekeeping	Yes/ Incentivised/ No

Type of PHC practice	Single-handed PHC practice (with/without nurse)<br>Multiple-handed practice (with/without nurse)<br>Multiple handed practice with nurse working as fully integrated team (number physician=nurse)<br>Any option above plus other specialists<br>Others
Pick and rank the most common type of patients seen in PHC practices	Covered under general taxation funding<br>Covered under SHI fund(s)<br>Entitled to public PHC due to low income<br>Private patient without insurance<br>Covered under PHI<br>Others
Co-payments	Yes/ Only prescriptions, diagnostic tests / No
Provider's workload drivers	Clinical practice/patients' visits<br>Skills and competence maintenance activities<br>Bureaucratic clinical tasks<br>Bureaucratic management tasks<br>Others
Reasons for prescribing outside the guidelines	Patients' pressure<br>Lack of knowledge/insufficient training<br>Lack of standardised guidelines<br>PHI requirements<br>Pharmaceutical companies' representatives<br>Local/regional/national authority constraints
**Round 2**	
**Variable**	**Categories**
Health system financing mechanism	National health system (NHS) (fund raised through a percentage of the general taxation collected from nation-wide citizens)<br>Compulsory national health insurance (CNHI) (fund raised through earmarked health care contributions collected from nation-wide citizens)<br>SHI managed with a single fund (raised through earmarked health care contributions collected from the workforce)<br>SHI managed through several funds (raised through earmarked health care contributions collected from the workforce into different funds according to professional types)<br>Voluntary national health insurance (fund raised from earmarked health care contributions gathered from citizens on voluntary basis)<br>Private health insurance (PHI) (funds raised through earmarked health care contributions gathered from individuals on voluntary basis into different funds according to insurer)<br>Individual out of pocket expenditure (no insurance model, each one pays what they consume)
PHC practice ownership	Government (national, regional or local) owns and manages facilities<br>Government (national, regional or local) owns facilities but public employees manage them<br>Government owns the facilities, but they are rented and managed by self-employed PHC physicians/professionals<br>Government owns the facilities, but they are rented and managed by private non-professional actors<br>Single or group of PHC physicians own and manage the facilities<br>Private (non-professional) actors own and manage the facilities<br>Others
Institution regulating providers' competences/skills maintenance regulation	Government regulation<br>Government-professional network regulation<br>Professional regulation<br>Private actors' regulation<br>No competences/skills maintenance regulation<br>Others
Institution regulating providers' clinical practice regulation	Government regulation<br>Government- professional network regulation<br>Professional regulation<br>Private actors' regulation<br>No clinical practice regulation<br>Others
Institution regulating providers' clinical practice regulation	Government regulation<br>Government-professional network regulation<br>Professional regulation<br>Private actors' regulation<br>No regulation of license conferral/no license required<br>Others
