# Supplementary material for: The impact of governance in primary health care delivery: a systems thinking approach with a European panel
Source: Health Res Policy Syst. 2019 Jul 4;17:65. doi: 10.1186/s12961-019-0456-8 (PMC6609383; doi:10.1186/s12961-019-0456-8)
Supplement: Supplementary file 2 — Evolution of the framework through three Delphi rounds. (PDF 107 kb) [file 12961_2019_456_MOESM2_ESM.pdf]

Additional file 2. Evolution of the framework through three Delphi round

Start → End (horizontal); Start ↓ End (vertical)

FIRST ROUND	SECOND ROUND	THIRD ROUND	FINAL FRAMEWORK
**Initial review (selected)**<br>(1–38)	**Review suggested by first round findings**<br>- Structure impact in delivery: employment status,(39,40) payments,(41,42) regulation,(43,44) ownership (45)<br>- Interrelations between structural functions and actors(22,46)	**Review suggested by second round findings**<br>- Interrelations between structural functions and actors: employment status, payments, regulation, ownership as governing instruments.(17,41,47–49)	**GOVERNANCE**<br>(governing actors/health system financing context)<br>↓
**INITIAL FRAMEWORK**<br><br>**Health system financing/ Governing actors**<br>**Financing**: Provision (staff, facilities); Reception (entitlements)<br>**Regulation**: Provision (staff, facilities); Reception (demand)	**REDEFINED FRAMEWORK 1**<br><br>**Health system financing/ Governing actors**<br>**Financing supply**: Employment status; Payments; Ownership<br>**Regulation supply**: Clinical practice; Competences License; Training; Facilities equipment; Facilities distribution	**REDEFINED FRAMEWORK 2**<br><br>**Health system financing/Governing actors**<br>**EXERCISE OF SUPPLY GOVERNANCE**<br>**Financing**: Employment status; Ownership<br>**Regulation**: Payment (reg. mechanism); Clinical practice; Competences, License; Training; Facilities equipment, distribution	**Implementation of Governance**<br>(who and how?)<br>**Financing** / **Regulation**<br>Providers — Financing: Employment status; Payments; Ownership — Regulation: Competences/ skills; Clinical practice; Training<br>Receivers — Financing: Entitlements; Co-payments — Regulation: Gatekeeping
**First round findings**<br>Structure impact in delivery: agreement on employment status, payments, ownership, entitlements, regulation and specialist training.<br>Interrelations between structural functions and actors:<br>-Entitlements (demand) and payment/employment status (supply).<br>- Regulation (competence and clinical practice) and financing.<br>- Health system financing (actors): strongly correlated to PHC functions.	**Second round findings**<br>Structure impact in delivery: reaffirm aspects<br>Interrelations between structural functions and actors:<br>- Providers' payments and regulation.<br>- Other functions interrelations (e.g., employment status/ownership, such as civil servant/public facilities)<br>- Actors involved (e.g., employment status and clinical practice regulation/accountability) determine functions (e.g., self-employed accountable to professional association)<br>- Employment status, payment, ownership, regulation: Exercise of Governance.	**Third round findings**<br>Consensus on:<br>- Structural functions impact delivery process.<br>- Structural functions: different if different actors involved (actors shape functions), this may affect delivery differently.<br>- Employment status, ownership, regulation: Implementation of Governance.<br>- Payments: regulatory mechanism.<br>- Need to include demand information: entitlements, co-payments, gatekeeping.	

Figure 1 Evolution of the framework through three Delphi rounds

The impact of governance in primary health care delivery: a systems thinking approach with a European panel

Authors: Ana Belén Espinosa-González, Brendan C Delaney, Joachim Marti, Ara Darzi

Figure 1 (above) illustrates the evolution of the framework throughout the Delphi process. As mentioned in the main text, the development of the framework involved an iterative process consisted of (1) literature review informing the framework, (2) survey with questions based on the framework and the review, and (3) analysis of the answers referring to panellists' countries PHC characteristics. The redefinition of the framework was supported by panellists' answers, correlations and literature. After the rounds, a video presentation feedback was sent to panellists for their review.

References that informed the development of the framework

1. Starfield B. Measuring the attainment of primary care. J Med Educ. 1979;54:361–9.
2. Shi L, Starfield B, Xu J. Validating the adult primary care assessment tool. J Fam Pract. 2001;50(2).
3. Esping-Andersen G. The Three Worlds of Welfare Capitalism. Princeton University Press. Princeton: Princeton University Press; 1990.
4. Schieber GJ, Poullier JP. Overview of international comparisons of health care expenditure. In: OECD, editor. Health Care Systems in Transition: The Search for Efficiency. Paris: OECD; 1990. p. 9–15.
5. Watson DE, Broemeling A-M, Reid RJ, Black C. A Results-Based Logic Model for Primary Health Care. Vancouver; 2004.
6. Murray J, Saxena S, Millett C, Curcin V, de Lusignan S, Majeed A. Reductions in risk factors for secondary prevention of coronary heart disease by ethnic group in south-west London: 10-year longitudinal study (1998-2007). Fam Pract [Internet]. 2010;27(4):430–8. Available from: http://ovidsp.ovid.com/ovidweb.cgi?T=JS&PAGE=reference&D=med5&NEWS=N&AN=20538744
7. Londoño JL, Frenk J. Structured pluralism: towards an innovative model for health system reform in Latin America. Health Policy (New York). 1997;41(1):1–36.
8. Roemer MI. National health systems throughout the world. Annu Rev Public Health [Internet]. 1993 [cited 2017 Aug 25];14:335–53. Available from: http://www.annualreviews.org/doi/pdf/10.1146/annurev.pu.14.050193.002003
9. Marmor TR, Okma KGH. Cautionary lessons from the West : what (not) to learn from other countries' experiences in the financing and delivery of health care. In: The state of social welfare, 1997 : international studies on social insurance and retirement, employment, family policy and health care. 1998.
10. Rico A, Saltman RB, Boerma WGW. Organizational restructuring in European health systems: the role of primary care. Soc Policy Adm. 2003;37(6).
11. WHO. Health Systems: Improving Performance [Internet]. Vol. 78. Geneva; 2000. Available from: http://www.who.int/whr/2000/en/whr00_en.pdf
12. Murray CJL, Frenk J. A framework for assessing the performance of health systems. Bull World Health Organ [Internet]. 2000 [cited 2017 Aug 16];78(6):717–31. Available from: http://www.ncbi.nlm.nih.gov/pubmed/10916909%5Cnhttp://www.pubmedcentral.nih.gov/articlerender.fcgi?artid=PMC2560787
13. European Commission. Euprimecare - Quality and costs of primary care in Europe. 2011;(241595). Available from: http://ec.europa.eu/research/health/public-health/health-systems/projects/euprimecare_en.html
14. Hsiao W. What is a health system? Why should we care? [Internet]. Harvard T.H. Chan School of Public Health. Cambridge; 2003. Available from: http://lingli.ccer.edu.cn/ahe2012/%5CWeek3%5CHsiao2003.pdf
15. Roberts M, Hsiao W, Berman P, Reich M. Getting Health Reforms Right: A Guide to Improving

Performance and Equity. Oxford University Press; 2008.

16. Mossialos E, Allin S, Figueras J. Health Systems in Transition: Template for analysis [Internet]. Copenhagen; 2007 [cited 2017 Aug 25]. Available from: http://www.wm.tu-berlin.de/fileadmin/a38331600/2007.publications/2007.Allin.Busse_Health.Systems.in.Transition.Template.for.analysis.pdf

17. Crampton P, Starfield B. A case for government ownership of primary care services in New Zealand: weighing the arguments. Int J Heal Serv. 2004;34(4):709–27.

18. Atun R, Menabde N. Health systems and systems thinking. In: Coker R, Atun R, Mckee M, editors. Health Systems and the Challenge of Communicable Diseases. Maidenhead: Open University Press; 2008.

19. Wendt C. Mapping European healthcare systems: a comparative analysis of financing, service provision and access to healthcare. J Eur Soc Policy. 2009;19(5):432–45.

20. Wendt C, Frisina L, Rothgang H. Healthcare system types: A conceptual framework for comparison. Soc Policy Adm. 2009;43(1):70–90.

21. Böhm K, Schmid A, Götze R, Landwehr C, Rothgang H. Five types of OECD healthcare systems: empirical results of a deductive classification. Health Policy (New York) [Internet]. 2013;113:258–69. Available from: http://dx.doi.org/10.1016/j.healthpol.2013.09.003

22. de Savigny D, Adam T, editors. Systems Thinking for Health Systems Strengthening. Alliance for Health Policy and Systems Research, World Health Organisation. Geneva: World Health Organization; 2009.

23. Siddiqi S, Masud TI, Nishtar S, Peters DH, Sabri B, Bile KM, et al. Framework for assessing governance of the health system in developing countries: Gateway to good governance. Health Policy (New York). 2009;90(1):13–25.

24. Rodwell J, Gulyas A. A taxonomy of primary health care practices: an avenue for informing management and policy implementation. Aust J Prim Health [Internet]. 2013;19(3):236–43. Available from: http://ovidsp.ovid.com/ovidweb.cgi?T=JS&PAGE=reference&D=medl&NEWS=N&AN=22950950

25. Brinkerhoff DW, Bossert TJ. Health Governance: Concepts, Experience, and Programming Options. Usaid [Internet]. 2008;(February):1–32. Available from: http://www.healthsystems2020.org/content/resource/detail/1914/

26. Mikkelsen-Lopez I, Wyss K, de Savigny D. An approach to addressing governance from a health system framework perspective. BMC Int Health Hum Rights [Internet]. 2011;11. Available from: http://bmcinthealthhumrights.biomedcentral.com/articles/10.1186/1472-698X-11-13

27. Sibthorpe B, Gardner K. A Conceptual framework for performance assessment in primary health care. Aust J Prim Health. 2007;13(2).

28. Hogg W, Rowan M, Russell G, Geneau R, Muldoon L. Framework for primary care organizations: the importance of a structural domain. Int J Qual Heal Care. 2008 Oct;20(5):308–13.

29. Bourgueil Y, Marek A, Mousques J. Three models of primary care organisation in Europe, Canada, Australia and New Zealand. Quest d'economie la Sante. 2009;141(April):1.

30. Polluste K, Kasiulevicius V, Veide S, Kringos DS, Boerma W, Lember M. Primary care in Baltic countries: a comparison of progress and present systems. Health Policy (New York) [Internet]. 2013;109(2):122–30. Available from: http://ovidsp.ovid.com/ovidweb.cgi?T=JS&PAGE=reference&D=medl&NEWS=N&AN=22964369

31. Szecsenyi J, Campbell S, Broge B, Laux G, Willms S, Wensing M, et al. Effectiveness of a quality-improvement program in improving management of primary care practices. CMAJ [Internet]. 2011;183(18):E1326-33. Available from: http://www.scopus.com/inward/record.url?eid=2-s2.0-83455236537&partnerID=tZOtx3y1

32. Schäfer WL, Boerma WG, Kringos DS, De Maeseneer J, Gress S, Heinemann S, et al. QUALICOPC, a

multi-country study evaluating quality, costs and equity in primary care. BMC Fam Pr [Internet]. 2011;12((Schäfer W.L.) NIVEL, Netherlands Institute for Health Services Research, Utrecht, The Netherlands.):115. Available from: http://www.embase.com/search/results?subaction=viewrecord&from=export&id=L560028660%5Cnhttp://sfx.library.uu.nl/utrecht?sid=EMBASE&issn=14712296&id=doi:&atitle=QUALICOPC,+a+multi-country+study+evaluating+quality,+costs+and+equity+in+primary+care.&stitle=B

33. Abimbola S, Negin J, Jan S, Martiniuk A. Towards people-centred health systems: a multi-level framework for analysing primary health care governance in low-and middle-income countries. Health Policy Plan. 2014;29:29–39.

34. Cleary SM, Molyneux S, Gilson L. Resources, attitudes and culture: an understanding of the factors that influence the functioning of accountability mechanisms in primary health care settings. BMC Health Serv Res [Internet]. 2013 [cited 2018 Mar 27];13:320. Available from: http://www.pubmedcentral.nih.gov/articlerender.fcgi?artid=3844434&tool=pmcentrez&rendertype=abstract

35. Donabedian A. Evaluating the quality of medical care. Milbank Mem Fund Q. 1966;44(3):166–203.

36. Field MG. The concept of "health system" at the macrosociological level. Soc Sci Med. 1973;7:763–85.

37. Terris M. The three world systems of medical care: trends and prospects. Am J Public Health. 1978;68(11):1125–31.

38. Frenk J, Donabedian A. State intervention in medical care: types, trends and variables. Health Policy Plan. 1987;2(1):17–31.

39. Goddard M, Gravelle H, Hole A, Marini G. Where did all the GPs go? Increasing supply and geographical equity in England and Scotland. J Heal Serv Res Policy. 2010;15(1):28–35.

40. Kinouani S, Boukhors G, Luaces B, Durieux W, Cadwallader J, Aubin-auger I, et al. Private or salaried practice : how do young general practitioners make their career choice ? A qualitative study. BMC Med Educ [Internet]. 2016;1–10. Available from: http://dx.doi.org/10.1186/s12909-016-0754-6

41. Robinson JC. Theory and practice in the design of physician payment incentives. Milbank Q. 2001;79(2):149–177, III.

42. Gosden T, Forland F, Kristiansen IS, Sutton M, Leese B, Giuffrida A, et al. Impact of payment method on behaviour of primary care physicians: a systematic review. J Health Serv Res Policy [Internet]. 2001;6(1):44–55. Available from: http://www.ncbi.nlm.nih.gov/pubmed/11219360

43. Scrivens. The future of regulation and governance. J R Soc Promot Health. 2007;127(2).

44. Nouwens E, van Lieshout J, Wensing M. Determinants of impact of a practice accreditation program in primary care: a qualitative study. BMC Fam Pract [Internet]. 2015;16:78. Available from: http://www.pubmedcentral.nih.gov/articlerender.fcgi?artid=4490740&tool=pmcentrez&rendertype=abstract

45. Russell G, Dahrouge S, Tuna M, Hogg W, Geneau R, Gebremichael G. Getting it all done. Organizational factors linked with comprehensive primary care. Fam Pract [Internet]. 2010;27(5):535–41. Available from: http://ovidsp.ovid.com/ovidweb.cgi?T=JS&PAGE=reference&D=med5&NEWS=N&AN=20534791

46. Prashanth NS, Marchal B, Devadasan N, Kegels G, Criel B. Advancing the application of systems thinking in health: a realist evaluation of a capacity building programme for district managers in Tumkur, India. Heal Res Policy Syst [Internet]. 2014 [cited 2017 Jul 22];12. Available from: http://www.health-policy-systems.com/content/12/1/42

47. World Health Organization. Everybody business: strengthening health systems to improve health outcomes: WHO's framework for action. [Internet]. Geneva; 2007 [cited 2017 Aug 16]. Available from: http://www.who.int/healthsystems/strategy/everybodys_business.pdf

48. Lewis M, Pettersson GG. Governance in Health Care Delivery [Internet]. Policy Research Working Papers. 2009 [cited 2017 Aug 16]. Available from: http://documents.worldbank.org/curated/en/792741468330936271/pdf/WPS5074.pdf

49. Sheaff R, Rogers A, Pickard S, Marshall M, Campbell S, Sibbald B, et al. A subtle governance: “soft” medical leadership in English primary care. Sociol Health Illn. 2003;25(5):408–28.
