# Supplementary material for: The impact of governance in primary health care delivery: a systems thinking approach with a European panel
Source: Health Res Policy Syst. 2019 Jul 4;17:65. doi: 10.1186/s12961-019-0456-8 (PMC6609383; doi:10.1186/s12961-019-0456-8)
Supplement: Supplementary file 3 — Correlations between PHC characteristics of Delphi panel countries. (PDF 176 kb) [file 12961_2019_456_MOESM3_ESM.pdf]

The impact of governance in primary health care delivery: a systems thinking approach with a European panel

Authors: Ana Belén Espinosa-González, Brendan C Delaney, Joachim Marti, Ara Darzi

Additional file 3. Correlations between PHC characteristics of Delphi panel countries

Table 1 Correlations table

		FM	FFM	MES	ES	ESS	PC	PCS	PM	MPM	PMS	O	OS	G	CR	CPR	LC	C	E
**FM**	**PC**	1	0.706**	0.303	-0.293	.410*	-0.303	0.103	-0.260	0.254	0.251	.528**	.592**	0.314	0.341	.519**	.493*	0.010	0.309
	**Sig**		0.000	0.150	0.164	0.047	0.150	0.632	0.221	0.232	0.237	0.008	0.002	0.136	0.103	0.009	0.014	0.962	0.141
**FFM**	**PC**	0.706**	1	0.208	-0.117	0.277	-0.250	0.120	-0.197	0.280	0.197	0.312	0.161	.408*	0.302	.421*	0.237	-0.073	0.125
	**Sig**	0.000		0.331	0.587	0.189	0.239	0.577	0.357	0.185	0.355	0.138	0.452	0.048	0.152	0.040	0.266	0.734	0.561
**MES**	**PC**	0.303	0.208	1	-0.160	.671**	.471*	0.290	0.057	.414*	0.150	.591**	.429*	.494*	0.076	0.099	0.270	-0.167	-0.216
	**Sig**	0.150	0.331		0.456	0.000	0.020	0.169	0.792	0.045	0.484	0.002	0.036	0.014	0.722	0.644	0.202	0.436	0.311
**ES**	**PC**	-0.293	-0.117	-0.160	1	-0.084	-0.097	0.187	0.071	-0.047	-0.287	-0.307	-0.207	-0.019	-0.206	-0.160	-0.228	-.582**	-0.102
	**Sig**	0.164	0.587	0.456		0.696	0.651	0.381	0.741	0.829	0.174	0.144	0.332	0.928	0.333	0.454	0.285	0.003	0.636
**ESS**	**PC**	.410*	0.277	.671**	-0.084	1	0.071	0.287	-0.224	.407*	0.164	.594**	.447*	0.364	0.000	0.068	0.213	-0.142	-0.060
	**Sig**	0.047	0.189	0.000	0.696		0.741	0.174	0.293	0.049	0.443	0.002	0.029	0.080	1.000	0.751	0.317	0.508	0.782
**PC**	**PC**	-0.303	-0.250	.471*	-0.097	0.071	1	.525**	.437*	.438*	0.359	0.192	0.046	0.073	-0.140	-0.310	-0.230	0.159	-.466*
	**Sig**	0.150	0.239	0.020	0.651	0.741		0.008	0.033	0.032	0.085	0.368	0.831	0.736	0.515	0.140	0.280	0.458	0.022
**PCS**	**PC**	0.103	0.120	0.290	0.187	0.287	.525**	1	0.371	.744**	0.402	.465*	0.367	0.157	0.144	0.238	-0.205	-0.009	-0.335
	**Sig**	0.632	0.577	0.169	0.381	0.174	0.008		0.074	0.000	0.052	0.022	0.078	0.463	0.501	0.262	0.336	0.968	0.109
**PM**	**PC**	-0.260	-0.197	0.057	0.071	-0.224	.437*	0.371	1	0.289	.471*	-0.017	0.168	-0.084	-0.034	0.008	-0.335	0.038	-0.019
	**Sig**	0.221	0.357	0.792	0.741	0.293	0.033	0.074		0.171	0.020	0.938	0.432	0.695	0.876	0.972	0.110	0.859	0.928
**MPM**	**PC**	0.254	0.280	.414*	-0.047	.407*	.438*	.744**	0.289	1	.465*	.538**	0.292	0.287	0.055	0.187	-0.054	0.066	-0.175
	**Sig**	0.232	0.185	0.045	0.829	0.049	0.032	0.000	0.171		0.022	0.007	0.166	0.174	0.800	0.382	0.804	0.758	0.414
**PMS**	**PC**	0.251	0.197	0.150	-0.287	0.164	0.359	0.402	.471*	.465*	1	0.331	0.350	-0.098	0.218	0.221	-0.064	0.397	-0.123
	**Sig**	0.237	0.355	0.484	0.174	0.443	0.085	0.052	0.020	0.022		0.114	0.094	0.648	0.306	0.300	0.766	0.055	0.567
**O**	**PC**	.528**	0.312	.591**	-0.307	.594**	0.192	.465*	-0.017	.538**	0.331	1	.797**	0.336	.481*	.418*	0.250	-0.064	-0.129
	**Sig**	0.008	0.138	0.002	0.144	0.002	0.368	0.022	0.938	0.007	0.114		0.000	0.108	0.017	0.042	0.238	0.766	0.547

**OS**	**PC**	.592**	0.161	.429*	-0.207	.447*	0.046	0.367	0.168	0.292	0.350	.797**	1	0.161	.468*	.412*	0.288	-0.162	0.141
	**Sig**	0.002	0.452	0.036	0.332	0.029	0.831	0.078	0.432	0.166	0.094	0.000		0.454	0.021	0.045	0.172	0.449	0.512
**G**	**PC**	0.314	.408*	.494*	-0.019	0.364	0.073	0.157	-0.084	0.287	-0.098	0.336	0.161	1	0.150	0.137	-0.147	0.036	-0.006
	**Sig**	0.136	0.048	0.014	0.928	0.080	0.736	0.463	0.695	0.174	0.648	0.108	0.454		0.484	0.522	0.492	0.866	0.979
**CR**	**PC**	0.341	0.302	0.076	-0.206	0.000	-0.140	0.144	-0.034	0.055	0.218	.481*	.468*	0.150	1	.771**	0.294	-0.061	0.038
	**Sig**	0.103	0.152	0.722	0.333	1.000	0.515	0.501	0.876	0.800	0.306	0.017	0.021	0.484		0.000	0.163	0.778	0.861
**CPR**	**PC**	.519**	.421*	0.099	-0.160	0.068	-0.310	0.238	0.008	0.187	0.221	.418*	.412*	0.137	.771**	1	.463*	-0.012	-0.002
	**Sig**	0.009	0.040	0.644	0.454	0.751	0.140	0.262	0.972	0.382	0.300	0.042	0.045	0.522	0.000		0.023	0.957	0.993
**LC**	**PC**	0.493*	0.237	0.270	-0.228	0.213	-0.230	-0.205	-0.335	-0.054	-0.064	0.250	0.288	-0.147	0.294	.463*	1	-0.238	0.273
	**Sig**	0.014	0.266	0.202	0.285	0.317	0.280	0.336	0.110	0.804	0.766	0.238	0.172	0.492	0.163	0.023		0.263	0.197
**C**	**PC**	0.010	-0.073	-0.167	-.582**	-0.142	0.159	-0.009	0.038	0.066	0.397	-0.064	-0.162	0.036	-0.061	-0.012	-0.238	1	-0.091
	**Sig**	0.962	0.734	0.436	0.003	0.508	0.458	0.968	0.859	0.758	0.055	0.766	0.449	0.866	0.778	0.957	0.263		0.672
**E**	**PC**	0.309	0.125	-0.216	-0.102	-0.060	-.466*	-0.335	-0.019	-0.175	-0.123	-0.129	0.141	-0.006	0.038	-0.002	0.273	-0.091	1
	**Sig**	0.141	0.561	0.311	0.636	0.782	0.022	0.109	0.928	0.414	0.567	0.547	0.512	0.979	0.861	0.993	0.197	0.672	

**. Pearson Correlation (PC) is significant (sig.) at the 0.01 level (2-tailed)
*. Pearson Correlation is significant at the 0.05 level (2-tailed)

Legend. FM: health system financing, FFM: fragmentation of financing mechanism, MES: main employment status, ES: type of employment status (aggregated), ESS: type of employment status (aggregated, simplified), PC: physician contract, PCS: physician contract simplified, PM: type of payment mechanisms (aggregated), MPM: main payment mechanism, PMS: type of payment mechanisms (aggregated, simplified), O: ownership, OS: ownership simplified, G: gatekeeping, CR: institution regulating physicians' competences, CPR: institution regulating physicians' clinical practice, LC: institution conferring license to practice, C: co-payments, E: patients' entitlements
