# Supplementary material for: The impact of governance in primary health care delivery: a systems thinking approach with a European panel
Source: Health Res Policy Syst. 2019 Jul 4;17:65. doi: 10.1186/s12961-019-0456-8 (PMC6609383; doi:10.1186/s12961-019-0456-8)
Supplement: Supplementary file 4 — Statements classified into governance/governing actors, financing and regulation functions. (PDF 160 Kb) [file 12961_2019_456_MOESM4_ESM.pdf]

The impact of governance in primary health care delivery: a systems thinking approach with a European panel

Authors: Ana Belén Espinosa-González, Brendan C Delaney, Joachim Marti, Ara Darzi

Additional file 4. Statements classified into governance/governing actors, financing ad regulation functions.

Table 1, **Table 2** and **Table 3** below show the level of agreement with the literature-based structure-process-outcome statements used to explore panellists' opinions on the potential impact of governance, regulation and financing in PHC delivery (provider and practice level) and outcomes. Tables are divided according to the main structural function (governance, regulation, financing) referred to in the statements. We also indicate the aspects of the PHC systems (within our Delphi panel countries) that correlate with agreement or disagreement to each statement and a summary of themes obtained after thematic analysis of panellists' comments. We provide two comments for each statement as an example. This information is summarised in the alluvial diagram (main text Figure 4).

Table 1 Statements referring to governance/governing actors

**Statement examples**	**Agree**	**Neither**	**Disagree**	**Agreement (A) or Disagreement (D) correlated to panel PHC background**	**Thematic analysis**
Round 1<br><br>1. For PHC physicians, the primary health care is their profession and source of income, it should not be considered as a public good, i.e., unlimited and freely available to all citizens	45·7%	13·6%	40·6%	A: Lack of clinical guidelines issued by institution holding accountability, lack of public employee physicians,<br><br>D: Contracts to national or local health authority	Misalignment of actors' interests, public responsibility, managerial behaviour, access.
	Comment example: "…the access to cultural goods, education, transportation etc should be free also..." (agree)<br><br>Comment example: "…I believe that a good and accessible PHC is a basic right in a modern moral society..." (disagree)				
Round 1<br><br>2. The objective of the PHC professionals as a collective is not necessary the same as the objective of the Ministry of Health (the health authority).	69·5%	8·5%	22%	SHI contracts, FFS and OOP payments, physicians' clinical practice and license regulated by professional institution; lack of guidelines issued by MoH and issued by institution holding accountability.	Alignment of actors involved, payment mechanisms, accountability, managerial behaviour.
	Comment example: "The Ministry may be more interested in saving money which could adversely affect patient care. As doctors, we must consider financial costs, but not if it means compromising health care" (agree)<br><br>Comment example: "It cannot be always the same, but I think the interests' alignment is good" (disagree)				
Round 1<br><br>3. As PHC physicians, we are a part of the health system, it is the health authority who should hold us accountable for our clinical practice.	57·7%	15·3%	27·1%	A: Civil servant physicians, lack of competences regulated by professional college.<br><br>D: SHI funds financing, FFS payments, lack of clinical practice regulated by the same institution they work for	Aims/objectives, public responsibility, regulator-regulated legitimacy, accountability, Cohesiveness, organisational - procedural justice.
	Comment example: "At the end State should be the best solution to protect population and organise health care" (agree)<br><br>Comment example: "Health authority could focus to much on spending rather than performance/quality of care" (disagree)				

Round 1<br><br>4. As PHC physicians we are part of a professional organisation, it is our professional body who should hold us accountable for our clinical practice.	66·1%	22%	11·9%	A: SHI contracts, lack of competences regulated by employer, entitlements due to PHI or OOP, competences regulated by professionals or private institution<br><br>D: Patients' entitlements due to low income,	Public responsibility, legitimacy, accountability
	Comment example: "Professional institution does not have authority to regulate physicians' clinical practice" (disagree)<br><br>Comment example: "It should be, but for a long time, any professional organisation makes this job and most of them don't care of the general interest" (agree)				
Round 1<br><br>5. When the number of PHC practices is insufficient for the population in a region, it is the PHC professionals' responsibility to accept the extra patients or develop new health centres	29%	16.9%	54.3%	D: Contracted to NHS, OOP payments, fixed salary, lack of competences regulated by licensing institution and of employer guidelines.	Public responsibility, ownership, payment mechanism, regulation of practice distribution, organisational justice, job satisfaction, access.
	Comment example: "This is the reality we have to face, although when the workload is high, this is not well accepted by GPs" (agree)<br><br>Comment example: "It is a government or health authorities' responsibility to provide care to extra patients and develop new health centres, not PHC physicians' responsibility" (disagree)				
Round 1<br><br>6. In some circumstances, e.g. the patient repeats the same consultation or explains economic difficulties (and is uninsured), physicians may not charge their patients for a consultation.	67.8%	15.3%	17%	A: OOP payments, PHI or OOP coverage,<br><br>D: Compulsory gatekeeping.	Public responsibility, actors' conflicts of interests, employment status, entitlements, managerial behaviour, access.
	Comment example: "This should not depend on the doctor" (disagree)<br><br>Comment example: "Being a "free profession" it is the physician's right to provide services according to his beliefs. I might not be a good idea (economically speaking) but it should be left for the physicians' discretion." (agree)				
Round 2<br><br>7. As PHC professionals, we must actively take part in the PHC policy development, especially in the decisions that affect management and physicians' practice' accountability.	94%	3·7%	1·9%	Physicians' owned facilities, lack of civil servants, FFS and OOP payments, competences regulated by government.	Inform policy development accountability, job satisfaction, organisational justice, policy acceptability and implementation.
	Comment example: "The professionals know what is needed in practice" (strongly disagree)<br><br>Comment example: "We do not have enough influence" (disagree)				
Round 2<br><br>8. Both the Ministry of Health and the PHC professionals have the common primary objective of improving the population health	87%	1·9%	11·2%	Public employees and government regulation of clinical practice correlates to strongly agreement. OOP and those who do not have competences regulated have lower agreement	Hierarchical actors, misalignment of interests, cohesiveness between actors. quality, costs, equality
	Comment example: "They should. Often PHC physicians advocate for their immediate population and not the regional or national population" (disagree)<br><br>Comment example: "... but they often do not appreciate this common objective on the same elements..." (agree)				
Round 2<br><br>10. When PHC physicians provide health care financed with public resources, their interventions should seek mostly to improve population health status instead of fulfilling patients' demands	50%	24%	26%	Having license conferred by institution holding accountability, co-payments per consultations, patients entitled to PHC due to low income.<br><br>Disagreement: FFS and OOP payments, contracts with SHI several funds, professionals' institution issuing license to practice.	Actors aims and interests, employment status and payment mechanisms, managerial behaviour.
	Comment example: "It's a good balance" (agree)<br><br>Comment example: "This is not a part of their contract. I still prefer individual health" (disagree)				

Table 2 Statements referring to PHC financing

<table>
<tr><th colspan="6">Agreement with statements referring to financing characteristics</th></tr>
<tr><th>Statement examples</th><th>Agree</th><th>Neither</th><th>Disagree</th><th>Agreement(A)/ Disagreement (D) correlated to panel PHC background</th><th>Thematic analysis</th></tr>
<tr><td rowspan="2">Round 1
1. Positive points of public health centres are that they all have the same equipment and provide similar services nationally, without requiring the PHC physicians being responsible for these.</td><td>37·3%</td><td>27·1%</td><td>35·6%</td><td>A: Physicians competences and clinical practice regulated by employer institution. MoH guidelines.
D: FFS, general taxation entitlements, Lack of competence regulation</td><td>Misalignment of actors' interests, regulation and monitoring, cohesiveness of actors, managerial behaviour, comprehensiveness, equality.</td></tr>
<tr><td colspan="5">Comment example: "Provided that the equipment is checked and changed based on a local control by PHC professionals" (agree)

Comment example:" Patients should have a real choice" (disagree)</td></tr>
<tr><td rowspan="2">Round 1
2. There is need for developing more health centres in our region but junior family physicians prefer working in existing centres and focus on clinical activities rather than setting up new practices to manage.</td><td>40·7%</td><td>28·8%</td><td>30·5%</td><td>A: Self-employed without contracts physicians.
D: Lack of FFS payments, lack of compulsory specialisation, lack of clinical practice regulation</td><td>Public responsibility, ownership, distribution regulation, access to PHC.</td></tr>
<tr><td colspan="5">Comment example: "Setting up practises and management are cited as a major burden that new GPs want to avoid"," Junior GPs don't want to practice alone in private sector" (agree)

Comment example: "Young physicians want to change practices and the existing health centres impose consultations duration that is too short to balance the books" (disagree)</td></tr>
<tr><td rowspan="2">Round 1
3. Public PHC physicians may provide health care less conscientiously than self-employed ones since their income does not depend on the number of patients they see or activities/results they achieve.</td><td>28·5%</td><td>23·7%</td><td>52·5%</td><td>A: Physicians' competences regulated by college.
D: Public ownership, lack of competence regulation, guidelines issued by accountability holding institution, employer institution and professional and public institutions.</td><td>Employment status, payment mechanisms, clinical and managerial behaviour, comprehensiveness continuity, access, quality, equality, costs.</td></tr>
<tr><td colspan="5">Comment example: "Self-employed physicians are more concerned in keeping patients on their lists and so may treat the patients according to their demands in order to "please" the patients" (disagree)

Comment example: "After 20 years of practice, this does not depend on the type of practice (private /public) but on the specific characteristics/personality of the GP" (agree)</td></tr>
<tr><td rowspan="2">Round 1
4.. Public PHC centres (provided and managed publicly) are worse equipped and maintained than private PHC practices.</td><td>37·3%</td><td>22%</td><td>40·7%</td><td>A: Contracted to one or several PHIs. Lack of competences regulated by employer.
D: guidelines developed by independent authority, lack of clinical practice regulated by central purchaser.</td><td>Actors governance, managerial behaviour, equipment regulation, comprehensiveness.</td></tr>
<tr><td colspan="5">Comment example: "Public PHC centres may try to save in all possible expenditures" (agree)

Comment example: "40 % of GPs are solo practice with poor budget for equipment" (disagree)</td></tr>
<tr><td rowspan="2">Round 1
5. When PHC physicians are in charge of equipping the facilities and arranging the services out of their budgets, i.e., paying the equipment or diagnostic tests for their patients or hiring staff, they may avoid some of those in order to save some expenditure.</td><td>56%</td><td>25·4%</td><td>18·7%</td><td>A: SHI single financing, private ownership, FFS payments, PHI or OOP entitlements, government guidelines, lack of compulsory gatekeeping.</td><td>Actors' interests, ownership, employment status, regulation, managerial and clinical behaviour, comprehensiveness, access.</td></tr>
<tr><td colspan="5">Comment example: "Self-employed GPs are contracted to insurance company and couldn't arrange anything out of contract" "If there are incentives to get equipment such as payments for doing ABPM. ECG etc, they will get the equipment" (disagree)

Comment example: "Self-employed GPs avoid basic equipment and especially renewable equipment they rarely make use of" (agree)</td></tr>
</table>

Round 1<br>6. PHC physicians paid on salary or capitation basis are not keen on seeing more patients outside their schedule in a busy day.	61%	11·9%	27·1%	A: Physicians' ownership, self-employed physicians, performance and FFS mixed payments, lack of guidelines issued by employer.<br>D: NHS financing types and guidelines issued by accountability holding institution.	Actors' interests and roles, payment mechanisms, managerial behaviour, access.
	Comment example: "Maybe not so keen but they do it because it is their duty" (disagree)<br><br>Comment example: "In our study, there are more patients without appointments in the group of independent contractors compared to PHC physicians who are working in public centres" (agree)				
Round 1<br>7. When physicians' income depends on the number of patients they see or services they provide, this may influence physicians' clinical practice and may lead to unnecessary therapies, tests or referrals.	67·8%	10·2%	22%	A: Compulsory national insurance financing, private ownership, MoH guidelines. Lack of competence and clinical practice regulation<br>D: contracted to local health authority	Actors' interests, professional ethics, physicians' competences and skills, payment mechanism, workload, access, quality, costs.
	Comment example: "If the physician is competent (professional ethics), this is not a problem" (disagree)<br><br>Comment example: "Especially when the workload increases (Friday evening, outbreaks period...)" (agree)				
Round 1<br>8. Presented with similar signs and symptoms, physicians may be more likely to adopt a "wait and see" approach with public patients than with private patients.	42·4%	18·6%	39%	A: Lack of institution regulating physicians' competences.<br>D: Guidelines issued by employer and by the same institution holding physicians accountable.	Employment status, payment mechanisms, entitlements, clinical behaviour.
	Comment example: "Antibiotics are prescribed more easily to private patients to keep them satisfied" (agree)<br><br>Comment example: "Tt probably depends more upon the physician's personality" (disagree)				
Round 1<br>9. Private patients' expectations for diagnostic and treatment activities can be high as they feel they are contributing more to the care they receive, and this may lead to unnecessary interventions or treatments	74·6%	13·6%	11·9%	A: Private ownership, lack of NHS contracts.<br><br>Level of agreement: OOP (no significant differences between subgroups)	Patients entitlements, physicians' competences, skills and ethics. Clinical and managerial behaviour, doctor-patient relationships. Access and costs.
	Comment example: "When patients go to doctors privately, it is almost as if the doctor has to give the patients their "money's worth" (agree)<br><br>Comment example: "It depends on the degree of its relationship with its personal physician and the level of its training" (disagree)				

Table 3 Statements referring to PHC regulation

**Agreement with statements referring to regulatory characteristics**					
**Statement examples**	**Agree**	**Neither**	**Disagree**	**Agreement(A)/ Disagreement (D) correlated to panel PHC background**	**Thematic analysis**
Round 1<br><br>1. When physicians are monitored on their clinical practice, the same organisation that monitors them should provide clinical guidelines to support them (in order to ensure some consistency between the guidelines and the clinical practice monitored!)	71·2%	10·2%	18·7%	A: PHI or OOP coverage, competence regulated, physicians' competences regulated by central or regional.<br><br>D: General taxation entitlements, lack of OOP or PHI entitlements, lack of competences regulation	Alignment, accountability, cohesiveness, adherence
	Comment example: "However, the guidelines must be done with physicians' approval or it will lead to a direct conflict…" (agree)<br><br>Comment example:" They should count on national guidelines" (disagree)				

Round 1<br><br>2. If the training of PHC physicians and the monitoring/regulation of their clinical practice are carried out by separate organisations (professional body and health authority), this can be inconsistent.	33·9%	30·5%	35·6%	A: Compulsory GP training,<br><br>D: Contract to regional NHS and SHI single fund, mixed payments, physicians' competence regulated by the same institution providing license, guidelines, competences regulated by same institution providing license, lack of competences regulated by regional authority and of competence regulation in general.	Regulation, consistency between training and monitoring, implementation and compliance, organisational justice
	Comment example: "There is a risk of contradiction and incoordination" (agree)<br><br>Comment example: "Training and monitoring imply different perspective" (disagree)				
Round 1<br><br>3.The coordination of PHC physicians with other specialists/hospital services can be difficult. Health authorities should establish clear links and pathways to make this coordination easier.	86.5%	5.1%	8.5%	A: Contracted to NHS or NHI, capitation and performance payment mixed.<br><br>D: SHI several funds financing, FFS payments	Regulation compliance, legitimacy, management.
	Comment example: "Clear and easy to follow" (agree)<br><br>Comment example: "This will generally increase the bureaucratic workload" (disagree)				
Round 2<br><br>4. PHC physicians' job is made still more difficult when the organisations in charge of their training, competence development and regulation of their clinical practice do not coordinate or agree.	92·6%	7·4%	0%	A: lack of physicians' competence regulation by professional college.<br><br>Type of institution issuing the guidelines	Regulator-regulated cohesiveness, compliance.
	Comment example: "One may require more than the possibilities provided by the others" (agree)<br><br>Comment example: "It sends conflicting signals to the health professionals" (agree)				
Round 2<br><br>5. The clinical practice of the self-employed PHC physicians should be regulated by the organisation they have a contract with (if any).	63%	14·8%	22·2%	A: fixed salary and capitation payments, employer guidelines.<br><br>D: PHIs and SHIs financing, contracted to SHI single fund, FFS or OOP, lack of gatekeeping, PHIs and OOP entitlements, lack of government regulation of competences, physicians' ownership.	Actors objectives, public responsibility, consistency of regulatory actors, accountability, managerial behaviour, costs, quality.
	Comment example: "It`s inevitable if this organisation is the one that provides the funds" (agree)<br><br>Comment example: "It should be regulated by public rules" (disagree)				
Round 2<br><br>6. Only the public employee PHC physicians should be regulated by the Ministry of Health / health authorities	77·8%	11·1%	11·1%	Disagreement with OOP, FFS, contracted to local/regional authority, competences regulated by licensing institution, central authority, lack of regulation.	Actors goals, public responsibility, regulator-regulated alignment, employment status. accountability, quality.
	Comment example: "Government, which issues licences to practice, should check that private GPs follow CME and keep up to date. Revalidation is important is this regard" (disagree)<br><br>Comment example: "There must be standardisation of Ministry of Health / health authorities" (agree)				
Round 2<br><br>7. Public planning of the distribution of PHC services can help decrease the inequalities in access to PHC in a country	88·9%	9·2%	1·9%	Civil servants, lack of FFS payments, type of institution conferring the license to practice.	Public responsibility, state responsibility, ownership governance, employment status, entitlements, financial (and others) incentives, accessibility, equality
	Comment example: "Self-organisation leads to less and less GPs working in deprived and poor area. Public health centres allowed to increase the number of GPs in those areas" (agree)<br><br>Comment example: "We must pay attention to freedom of exercise in case of public planning" (neither A/D)				

Round 2<br><br>8. When the development of PHC facilities is the physicians' responsibility, the Ministry of Health should be still responsible for ensuring adequate equipment and equal distribution of PHC across the country.	81.5%	13%	5.5%	NHS financing types, physicians' competences regulated by employer, license conferred by government-professional network.<br><br>Lack of: private actor ownership of facilities, SHI contracts and entitlements to access PHC.	State public responsibility, legitimacy, governing and financing actors' alignment, interests' alignment, physicians' employment status, financial incentives, facilities ownership, regulation, access, comprehensiveness, organisational justice, equality.
	Comment example: "It depends on how PHC system is funded, if PHC works within insurance system, this one also has specific requirements for equipment. Thus, there would be two institutions that regulate this aspect: the MoH when opening the facility and the NIH when contracting" (disagree)<br><br>Comment example: "Inevitably some physicians wouldn`t be able to provide as much necessary equipment as the others, which would put their patients in disadvantage" (agree)				
Round 2<br><br>9. When professional bodies develop clinical guidelines, these are not only based on the efficiency of the interventions recommended, but also on cost-effectiveness.	70·4 %	18·5%	11·1%	NHS financing, civil servants, capitation and performance mixed payments, license to practice conferred by government, clinical practice regulated by physicians' employer.<br><br>- Disagreement: PHI or OOP coverage, contracted to SHI funds.	Inclusion, interests' alignment, regulation, cohesiveness of actors, compliance and adherence, quality and costs.
	Comment example: "Professional bodies take efficiency of the interventions in focus, which sometimes is connected with cost effectiveness" (disagree)<br><br>Comment example: "Clinical guidelines must be practical and applicable. Ignoring the financial constraints could make such guidelines useless" (agree)				
Round 2<br><br>10. Clinical recommendations based on cost-effectiveness are meant to distribute the health care resources homogeneously among the population, prioritising population health over patient-centred health care.	55%	25·9%	18·5%	Disagreement in FFS and OOP payments, MH guidelines, guidelines issued by employer institution, PHI payments	Actors' objectives, interests' alignment, cohesiveness between actors.
	Comment example: "This may sometimes be the case, but it is hard for people to accept" (agree)<br><br>Comment example: "Saving of money is the first objective. A possible virtuous consequence could be the above statement....in a virtuous administration ..." (disagree)				
