# Supplementary material for: The impact of governance in primary health care delivery: a systems thinking approach with a European panel
Source: Health Res Policy Syst. 2019 Jul 4;17:65. doi: 10.1186/s12961-019-0456-8 (PMC6609383; doi:10.1186/s12961-019-0456-8)
Supplement: Supplementary file 5 — Framework described using unified modelling language notations. (PDF 659 kb) [file 12961_2019_456_MOESM5_ESM.pdf]

The impact of governance in primary health care delivery: a systems thinking approach with a European panel

Authors: Ana Belén Espinosa-González, Brendan C Delaney, Joachim Marti, Ara Darzi

Additional file 5. Framework described with unified modelling language notations

Modelling in health system has evolved in order to describe processes and generate generic models that allow comparisons between health systems (1). This is particularly useful when health systems differ in their structural elements. Unified modelling language (UML) is a general purpose modelling language (2,3) used in software engineering to describe the describe the systems and produce analysable frameworks. The UML tool enables the representations of the model with different diagrams, such as use case diagrams and class diagrams (4). In this study, we used the StarUML software publicly available on http://staruml.io/.

1. Use Case diagram type

The use case diagram is a type of dynamic diagram that aims at emphasising the actor that develops a particular function (3).

Use Case diagrams have four basic components: (1) system, identified as "something that performs a function"; (2) actors, described as "something that uses the system", this can be a person or another system (external to the system we are modelling); (3) use cases, which describe the action that a user (actor) takes at any given moment, for example, the regulator in our system will monitor providers using the regulatory system; and (4) relationships, which are illustrated with lines connecting actors to use cases.

The actors in Use Case diagrams have attributes (e.g., degree of decentralisation), which allows the description of the type of actor that is performing the function. Actors can also have operations, which are the functionalities they can perform. Use cases may reproduce specific functionalities identified within a health system function, for example, defining the regulatory framework, designing the mechanism to regulate, delegating it to other actors if needed (private, professionals, community), and devolving or fragmenting the execution of the function to other levels within the same system. An actor can be related to multiple use cases and use cases may be related to different actors. For example, PHC professionals provide health care, but also are regulated or trained by other actors, such as private or public regulatory institutions.

Figure 1 below depicts the framework using UML Use Case diagram notations. The health system contains PHC system. Governing actors in the health system influence PHC regulation and financing actors, which are also interrelated and both influence PHC provider and practice. This ultimately impacts outcomes.

2. Class diagram type

Class diagrams (figure 2) are useful to describe the main elements of a system and their interrelations. By representing our framework as a class diagram, each function (e.g., regulation) is a sub-system that is contained within a higher subsystem and a system (e.g., PHC subsystem and health system). Regulation consists of many classes that represent items or functionalities, for example, supply regulation and demand regulation. Each class is defined on its attributes (e.g., type of actors developing the function) and operations (e.g., particular aspect to regulate) (3). When attributes and operations acquire specific values, a class becomes an object or instance (e.g., public institution regulating physicians' competences). This enables the representation of the framework developed in our study and the illustration of the interrelations identified (included in Additional file 2).

References

Kumarapeli P, de Lusignan S, Koczan P, Jones B, Sheeler I. 2007. The feasibility of using UML to compare the impact of different brands of computer system on the clinical consultation. *Informatics in Primary Care* **15**: 245–53.

Lusignan S De, Mcnulty R, Krause P. 2016. Accessible modelling of complexity in health and associated data flows : asthma as an exemplar. **23**.

Roff JT. 2003. *UML A Beginner's Guide*. McGraw-Hill: Berkeley.

Vasilakis C, Lecnzarowicz D, Lee C. 2010. Application of Unified Modelling Language (UML) to the Modelling of Health Care Systems Article in. International Journal Of Healthcare Information Systems And Informatics.

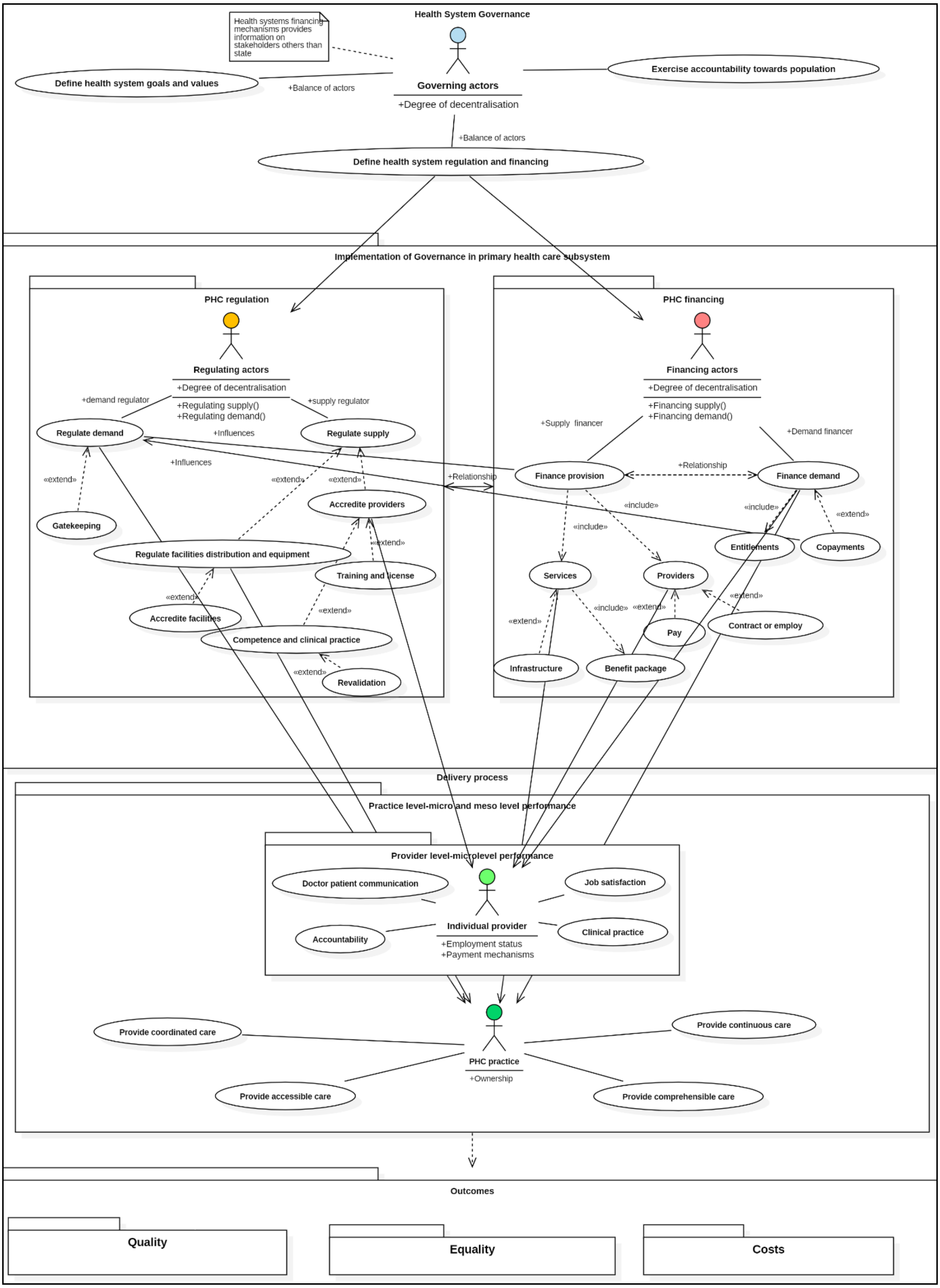

Figure 1 Final framework represented with UML Use Case diagram notations

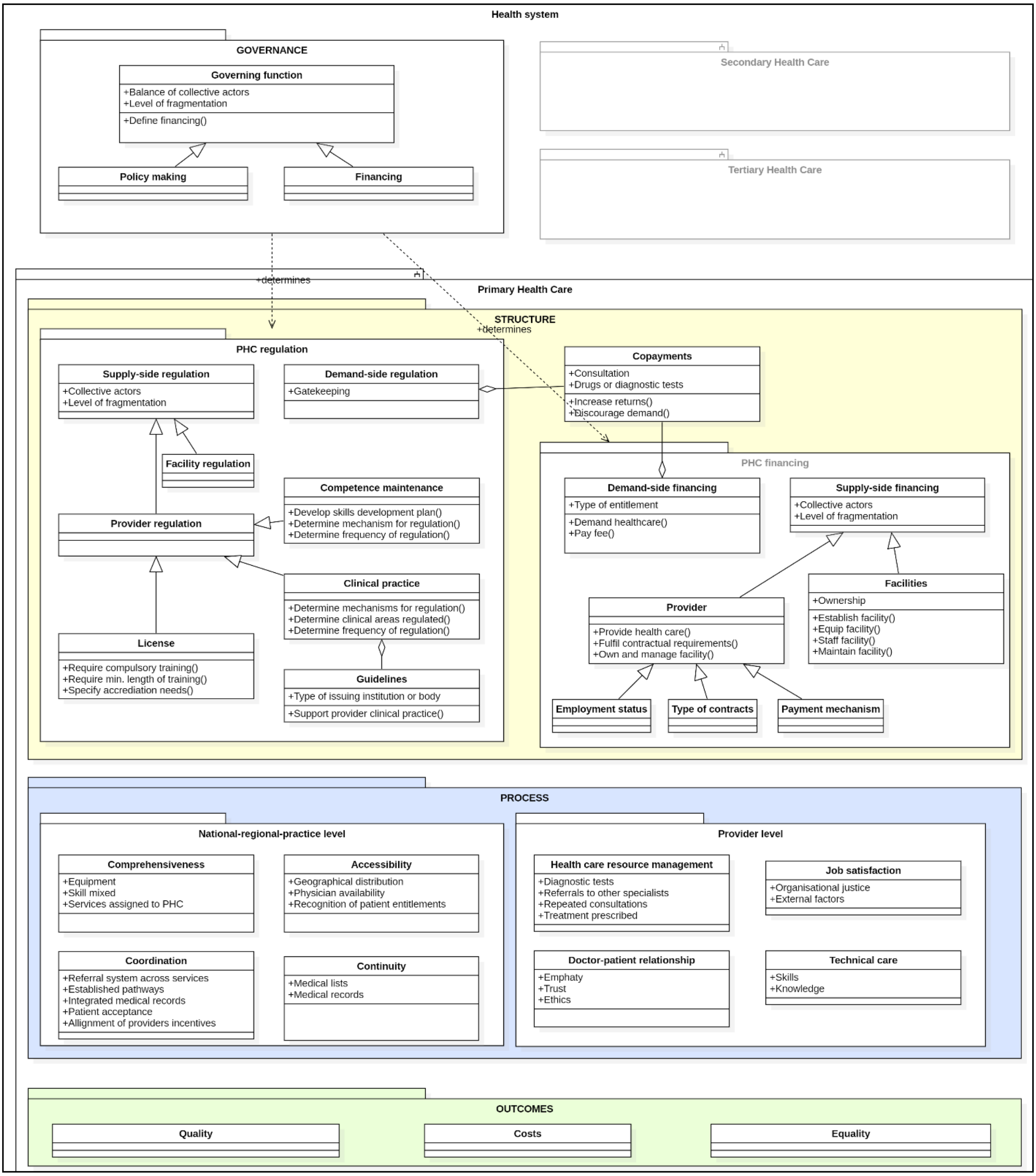

Figure 2 Final framework represented with UML Class diagram notations
